# Towards Evidence of Rigour in Empirical Deliberative Democratic Methods: Development and Piloting of the C‐JuRI Framework

**DOI:** 10.1111/hex.70608

**Published:** 2026-04-08

**Authors:** Emma K. Frost, Yves Saint James Aquino, Annette Braunack‐Mayer, Lucy Carolan, Stacy M. Carter

**Affiliations:** ^1^ Australian Centre for Health Engagement, Evidence and Values, School of Social Sciences, Faculty of the Arts, Social Sciences and Humanities University of Wollongong Wollongong Australia

## Abstract

**Background:**

Deliberative democratic methods are increasingly being used to involve the public in health policy decision‐making. These methods are rooted in deliberative democratic theory, which proposes the methods as democratic, inclusive and relevant ways to involve the public in policy decisions. Many practitioners have created methods for evaluating aspects of deliberative democratic engagement, but there are few practical guidelines for evaluating the quality of deliberative democratic processes as a whole. The aim of this study was to develop and pilot a framework for evaluating the quality of Citizens' Juries based on the OECD's *Evaluation Guidelines for Representative Deliberative Processes*.

**Methods:**

We developed the Citizens' Jury Rigour and Improvement (C‐JuRI) framework based on the OECD's criteria for evaluating deliberative democratic processes. We justified each criterion with respect to the theoretical literature on deliberative democracy. We describe the process of piloting the framework on the artificial intelligence (AI) in Healthcare Jury—the first national Citizens' Jury on using AI in healthcare.

**Results:**

The C‐JuRI framework was an effective tool for evaluating the AI in Healthcare Jury. Using the framework, we identified and reported on several strengths of the AI in Healthcare jury, as well as some opportunities to make changes to future processes to better work towards a deliberative democratic ideal. Post hoc additions to the framework may have assisted us in identifying more opportunities for improvement.

**Conclusion:**

The C‐JuRI framework is a useful tool for researchers and practitioners aiming to design high‐quality Citizens' Jury processes and for policymakers assessing the quality and rigour of deliberative democratic processes.

**Patient or Public Contribution:**

This framework is designed to evaluate the quality of initiatives to involve the public in decision‐making. The framework was piloted on a jury in which 28 members of the public deliberated about the use of AI in healthcare.

**Abbreviations**
AI: artificial intelligence
C‐JuRI: Citizens' Jury Rigour and Improvement Framework
OECD: Organisation for Economic Cooperation and Development
T1: Timepoint 1
T2: Timepoint 2
T3: Timepoint 3

## 1 Introduction

There are numerous reasons to argue that publics should be involved in creating the health policies that affect their lives. Public participation creates policies which reflect the views and interests of diverse people in the community [1, 2]. It works towards greater epistemic justice [3], by recognising publics as valued knowers and contributors to addressing normative problems. It also offers public recognition and respect [4] by respecting publics as people who deserve to have input into the rules that protect and govern them.

Whilst there are strong, and sometimes institutionalised, imperatives for public involvement in decision‐making [5], the practice of public involvement is neither simple nor uncontroversial. Reviews of empirical initiatives have found that there is little agreement on who ‘the public’ is, what is meant by ‘public involvement’, and what constitutes a successful public involvement initiative [6, 7]. This lack of clarity around what *good* public involvement looks like makes it difficult for researchers and decision‐makers alike to evaluate public engagement initiatives [8]. Moreover, political theorists have contended that not all publics have the necessary understanding of complex public policy issues to effectively participate in decision‐making initiatives [9]. They argue that politicised media and poor access to accurate information on policy issues can compromise the public's ability to contribute to an effective decision‐making process [2]. As a result, designers of public involvement initiatives must also contend with politicised information landscapes and theorists who doubt the public's ability to contribute to policy decisions at all.

Deliberative democracy theory engages with these critiques by providing a set of ideals for effective public involvement in policy decision‐making [10, 11]. The closer a process aligns with these ideals, the easier it becomes to argue that its outcomes represent the public's informed and considered views [12]. Fundamentally, deliberative democrats propose that a more rigorous process leads to more legitimate outcomes. Although the deliberative democratic ideals are well‐developed, there are few practical resources for understanding how empirical deliberative democratic processes should go about working towards those ideals.

Citizens' Juries are an increasingly common deliberative democratic method for public involvement in policy decision‐making. Our aim for this paper was to present a comprehensive framework for evaluating the rigour of Citizens' Juries, as a way to assess the legitimacy of their outputs. Ultimately, this paper responds to the question: *what conditions need to be met for deliberative democratic public involvement processes to be considered legitimate?* The paper is structured in three parts. The first part describes what a Citizens' Jury is, outlines the methodology's relationship to deliberative democratic theory, and provides a brief explanation of why an evaluative framework is necessary. The second part introduces our evaluative framework. The third part demonstrates how the framework can be used by presenting the results of our evaluation of our own Citizens' Jury on public views on artificial intelligence (AI) in healthcare.

### 1.1 Citizens' Juries and Deliberative Democracy

Citizens' Juries or Community Juries (henceforth referred to as *Juries*)^1^ are an increasingly popular method for involving publics in decision‐making [13]. Juries bring together a group of citizens (referred to as ‘jurors’), to learn about a specific issue from experts, deliberate on that issue, and produce justified recommendations [1]. Juries typically involve 25–35 participants who are randomly selected from a population, and who are broadly demographically representative of that population [14].

The rationale and justification for the Jury methodology is in the theory of deliberative democracy [13]. Deliberative democracy is based on the idea that policy should be guided by the informed and considered opinions of publics [15]. Deliberative democratic methods are based on the idea that policies should be informed by smaller‐scale processes which allow organisers to facilitate discussion, learning and reflection amongst all participants [9]. Deliberative democrats argue that, when given the information and time to learn about a policy problem, the public is able to make informed and rational judgements about a policy issue [9]. The output of a deliberative democratic process is a recommendation or series of recommendations that are said to represent what the broader public would recommend, if they had the opportunity to learn about and deliberate on the issue [9].

Although deliberative democratic methods are founded on appealing ideals about public involvement, the implementation of these ideals is complex. Deliberative democrats have been criticised for developing idealistic theories in lieu of engaging with the messiness of an empirical deliberative process [1].

Valuable practical evaluation methods for deliberative democratic processes do exist, but they are typically focused on evaluating a specific component of the process, rather than the entire process. Several of these methods involve analysing transcripts of the deliberations. Scott and colleagues [16] developed a deductive coding framework with a series of goals. The quality of the deliberation is assessed based on whether there is evidence in the transcripts that each of the goals is met. O'Doherty [12] also recommends the use of transcripts to evaluate deliberative quality. Instead of operationalising goals, O'Doherty recommends evaluating the extent to which jurors' statements are increasingly informed as the deliberations progress. In a good deliberative process, jurors may be uninformed at the beginning, but will move towards statements that consider a broader set of views and considerations. Similarly, Niemeyer and Veri [17] propose a Deliberative Reason Index (DRI) which examines the extent to which jurors justify their views with relevant reasons (coherence), acknowledge other arguments (reciprocity) and appeal to shared considerations (appropriateness). They use quantitative scoring to generate an overall DRI score to assess the quality of deliberations. These methods are valuable in assessing the quality of the deliberation, but they do not have a mechanism to evaluate other aspects of the process, such as the representativeness of the participants or the quality of the evidence shared.

Other practitioners have taken a different approach, using participant questionnaires to assess the participants' views on process quality. Timotijevic and Raats, Newson et al. and Simpson et al. each demonstrate the use of a participant questionnaire to identify the extent to which the participants felt that each aspect of the process was effective. These methods are effective at identifying potential opportunities for improvement, but they lack a mechanism to evaluate aspects of the process beyond the experiences of the participants.

Julia Abelson and the PPEET Collective [18] developed a thorough multi‐stage evaluation process for patient and public engagement methods. Their tool involves triangulating results from questionnaires administered to the participants, those planning the process, and project sponsors. Whilst this tool is comprehensive for evaluating patient and public engagement initiatives more broadly, it is not specific to processes based on deliberative democracy, and is not necessarily designed to assess the quality of Citizens' Juries against the deliberative democratic ideals.

In 2021, the OECD released guidelines for evaluating deliberative projects to address inconsistencies in the design of deliberative methods [13]. This guideline provides a set of concepts (‘criteria’) to guide the evaluation of the components of a deliberative process. This work is valuable, but the OECD guidelines provide little practical guidance on how to operationalise these concepts to evaluate a deliberative process.

In the following section, we describe how we build on the valuable frameworks developed by others, together with the OECD evaluation criteria, to develop a new framework for the evaluation of juries. Our aim was to present a comprehensive framework for evaluating the rigour of Citizens' Juries. First, we explicate the link between the OECD criteria and deliberative democracy theory's ideals. Then, we adapt the criteria into a framework comprised of assessable items. Finally, we demonstrate its use by evaluating a Citizens' Jury on AI in Healthcare.

## 2 Methods

### 2.1 Developing the Citizens' Jury Rigour and Improvement (C‐JuRI) Framework

In this section, we describe the development of the C‐JuRI framework. We have built on the OECDs [13] criteria in two ways. First, we have discussed and justified each of the criteria below by describing how it contributes to achieving a deliberative democratic ideal. Whilst the descriptions of the criteria provided by the OECD refer implicitly to theory, we wanted to make these connections explicit to connect deliberative democracy theory with the evaluative principles and Jury practice. Second, consistent with OECD recommendations, we have provided a practical framework for evaluating these criteria through three empirical means: a participant survey, a transcript analysis and a document review. Taken together, this paper is a comprehensive guide to what should be evaluated, how it can be evaluated and why it is important.

Whilst the OECD criteria are for evaluating deliberative democratic processes in general, the C‐JuRI framework is specifically targeted towards the evaluation of jury processes. Juries are distinctive as a deliberative method because they involve the recruitment of smaller cohorts of participants (typically 25–30) compared to other deliberative methods, which may have over 100 participants [1]. Recruiting smaller groups of participants creates both challenges and opportunities. On one hand, it potentially decreases the extent to which a cohort of people can be said to represent the broader population [1]. On the other hand, it gives organisers more insight into the dynamics of the deliberation. Jury small group sessions can feasibly be audio‐recorded, which can be leveraged as a resource to evaluate interactions between participants. To ensure the framework is granular enough to guide empirical projects, we have opted to make it specific to Juries.

We tested the framework on a Citizens' Jury on AI in Healthcare [19]. This Jury involved an online component where jurors heard information from experts over multiple weeks, followed by a 3‐day face‐to‐face meeting where jurors deliberated and formed recommendations. Some elements of this framework, particularly the timing of participant questionnaires, may need to be adjusted to fit other implementations of the Jury method.

This section describes our framework. First, we describe the criteria and relate them to deliberative democracy theory and ideals. Second, we describe the framework. The final section of this paper describes how the framework can be implemented to evaluate a Jury.

#### 2.1.1 Framework Criteria

We noted earlier that deliberative democrats have been criticised for developing ideals rather than practical resources for empirical projects. Whilst we agree that there is a wealth of ideals and a dearth of practical resources, we have found deliberative democratic ideals useful for anchoring empirical projects in deliberative democratic theory. We echo Bächtiger and colleagues' characterisation of these ideals as ‘aspirational qualit[ies]’ of empirical deliberative democratic work. They write: ‘[t]hat deliberative democracy in its ideal form cannot be achieved perfectly in the world of practice does not undermine its use as a standard towards which to strive’ [10]. Although no process can be ideal, we maintain that processes can be closer to or further away from the ideal. The further away the process is, the more it may warrant critical reflection about whether the conclusions could be considered deliberative (representing the considered views of participants) or democratic (representing the views of the broader public). Our framework provides users with a guide to evaluating the degree to which a Jury conforms to deliberative democratic ideals. We will explain these ideals more fully below.

The evaluative criteria proposed by the OECD fall into two categories. The first is ‘process design integrity', which contains criteria for evaluating the design of the deliberative process. The second is ‘deliberative experience’, which contains criteria for evaluating the effectiveness of the process. For the C‐JuRI framework, we followed the categories but made some minor adjustments to the criteria. The OECD criteria include ‘suitable design’. While we agree that design is important, we found this criterion was not sufficiently distinct from the other criteria to operationalise in a mutually exclusive way. To ensure design was addressed, we incorporated the OECD's Good Practice Principles [20] into the other criteria. We also changed the wording of the OECD criterion ‘clear and suitable purpose’ to ‘suitable purpose’ since clarity was addressed in ‘clear and unbiased framing’. In Table 1, we show the OECD's criteria and verbatim description on the left. On the right, we justify each criterion by appealing to deliberative democratic theory.

**Table 1:** OECD criteria and descriptions (adapted from [13]), and how each criterion helps an empirical process work towards a deliberative democratic ideal.

OECD criteria^a^	OECD description (verbatim)^a^	Our interpretation of how the criteria help a process move towards deliberative democratic ideals
Process design		
Suitable purpose	The deliberative process was commissioned for a suitable purpose, addressing a policy issue. (See Catching the Deliberative Wave report ([20]) Chapter 4, section Scope of the remit for guidance.) The mandate was clear, and it was clear how the recommendations would be used. The deliberative process was connected to the broader political system or policy‐making cycle.	An ideal deliberative democratic process is embedded within its political system. It encourages relevant decision‐makers to commit to responding to, or acting on, the jury recommendations [20], and it should create outputs that are contextually applicable and relevant for decision‐makers [9]. Ensuring that the remit (or question) addresses a policy‐relevant issue and that relevant decision‐makers are involved in the project leads to recommendations from the Jury that are more visible and usable for decision‐makers.
Clear and unbiased framing	The question addressed by the deliberative process was framed in a non‐leading, unbiased, clear way, easily understandable to the wider public.	For discussions between jurors to be considered ideally ‘deliberative’, they must be addressing a question that is free from any manipulative influence from stakeholders [1]. The organisers of a Jury are responsible for ensuring that the remit (or question) avoids framing the policy issue in a way that is likely to lead jurors towards certain solutions or recommendations. Recent literature on deliberative democracy has acknowledged that no process can be entirely unbiased or free of coercive influence [10]. In any process, the organisers and other stakeholders make decisions about issue framing that highlight or obscure certain elements of the process [21]. The goal is to ensure that the framing is appropriately broad and to minimise the influence of those with vested interests in the outcome.
Procedural design involvement	Organisers had an established process to call for, respond to, and recognise comments from stakeholders regarding the deliberative process design. A wide range of stakeholders representing diverse views had an opportunity to review the deliberative process design. Experts in the policy area were consulted over the questions and the choice of evidence provided. Deliberative democracy experts (in‐house or external) were consulted on process design.	Ideal deliberative democratic processes have both epistemic and political functions [9, 22]. Involving subject matter experts serves the epistemic function by ensuring that the information provided to participants is accurate and relevant, and that the process is not founded on inaccurate information [22]. Involving policy experts serves the political function by ensuring that the process is addressing a relevant policy question [20].
Transparency and governance	There were clear terms of reference, rules of engagement, codes of conduct, or ethical frameworks that governed the process. They were followed throughout the process. Information about the goals, design, governance of the process, funding source, civic lottery and any other materials was published publicly. The design of the process was free of external interference.	Deliberative democrats problematise the opacity of liberal democracy, which often has little accountability to real public interests [1]. Deliberative democratic processes, in contrast, are intended to be transparent and accountable to the citizenry [10]. Being accountable to external parties and making information about the process available online opens the process up to public and external scrutiny.
Representativeness and inclusiveness	Everyone had an equal opportunity, via a civic lottery, to be selected as a member of a deliberative process. (e.g., all residents or eligible voters.) The final group of members was a broadly representative sample of the general public (reflecting the demographic composition of a community, city, region, or country). (Anyone looking at the members could see ‘someone like me’ within the process.) Efforts were made to involve under‐represented groups. (In some instances, it is desirable to oversample certain demographics during the random sampling stage of recruitment to help achieve representativeness.) Efforts were made to remove barriers to participation. The OECD Good Practice Principles identify remuneration of the members, covering their expenses and/or providing or paying for childcare and eldercare as helpful ways to encourage and support participation.	Deliberative democratic processes are referred to as ‘microcosms’ [9] because they demonstrate deliberative decision‐making in a small sample of citizens. The citizens involved in a deliberative democratic process are expected to represent the views, values and experiences of the broader community [1]. The quality of deliberation is enhanced when there are inputs from all relevant views, values and experiences [23]. Representing all major demographic groups in the jury increases the chances that a diversity of views and experiences will be represented. Selecting a random and demographically representative sample of participants ensures that participation in the event is not limited to traditionally politically active individuals or groups [9]. Offering participants funding to pay for travel and childcare enables geographically remote and socioeconomically disadvantaged citizens the opportunity to participate. This makes the process more democratic because it ensures that everyone has an equal opportunity to participate.
Deliberative experience		
Neutrality and inclusivity of facilitation	The facilitation ensured inclusiveness, equal access to speaking opportunities and an appropriate balance of small group and panel discussions throughout deliberation. Enough consideration was given for marginalised communities to be heard. (e.g., via supportive and mindful facilitation, creating a safe space for expression, devising specific strategies for encouraging participation by those who are not used to speaking in public or who may feel intimidated.) Facilitation was neutral regarding the issue addressed.	Facilitation is necessary in deliberative democratic processes to keep participants on‐task and to ensure that all participants have an equal opportunity to contribute to discussions [10, 21]. However, facilitation is often described as a ‘balancing act’ [21] because, if facilitators are not mindful of their neutrality, they may inadvertently influence the deliberations by sharing their own views. The facilitator, in an ideal process, encourages equal participation without sharing their own personal views on the issue.
Accessible, neutral and transparent use of online tools	Any online tools used throughout the process were equally accessible to all members. There was assistance, training, equipment and internet connection offered and/or provided. (For some members who are unfamiliar with the internet or online tools, it may be necessary to have one‐on‐one support during the process.) The design of the online tools used was neutral and transparent (e.g., the algorithms or formulas used for preference or vote counting were explicit and clear, online tools ensured anonymity of members when needed, and the results calculated/aggregated using online tools were auditable).	Ensuring that all participants are able to access the online tools and content in a deliberative democratic project contributes to the inclusiveness of the process (see rationale for representativeness and inclusiveness). Neutrality and transparency of online tools serve a different function. The organisers of deliberative democratic processes are expected to be transparent about the process, which contributes to the legitimacy of the outcome through publicity [10, 20]. Technologies that may confuse or obfuscate the relationship between the process and its recommendations (e.g., a complex preferencing algorithm) could damage the legitimacy of the recommendations.
Breadth, diversity, clarity and relevance of the evidence and stakeholders	Members were provided a solid and accessible information base featuring a wide range of accurate, relevant, clear and accessible evidence and expertise, sufficient for effective participation and to address the remit set. The information base as a whole was neutral, with a breadth of diverse viewpoints represented. (Ensured, e.g., through mapping all the arguments of the issue with stakeholders to see whether all relevant areas and viewpoints are reflected in the information base.) The information base was accommodating to members with different learning styles and included materials in a variety of forms (written, video, in‐person expert presentations, etc.). There was a wide range of stakeholder views. (This could include an element of public submission.) The selection of sources was transparent, revealing the curator and the basis for selecting the content. People in charge of preparing the information base had declared any potential conflict of interest. Members had the possibility to submit evidence for consideration and request additional information.	Political philosophers often discredit the public's ability to participate in political forums due to their potential to be misinformed or uninformed about political problems [9]. Exposing participants to correct, diverse and balanced information about the policy issue in a deliberative democratic process encourages deliberation that is based on the best evidence available [24]. Aside from contributing to the epistemic function of the deliberative process, the provision of information to participants in a deliberative process also contributes to the inclusivity function. More educationally privileged participants are likely to enter a deliberative democratic process with higher levels of knowledge. Providing information to all participants works towards the ideal of inclusivity by providing equal access to information for all participants [9]. Practitioners have highlighted the importance of making this information as diverse and accessible as possible to enhance comprehensibility [25, 26]
Quality of judgement	There was consideration of conflicting values and structural issues underlying the question at hand. There was an emphasis on diversity of viewpoints, weighing of alternatives and trade‐offs, exploring uncertainties and exposing assumptions. Members provided justifications for their viewpoints. Members approached the process with open‐mindedness. Members considered and integrated a range of evidence in their judgements.	In contrast to other forms of democracy, deliberative democracy requires *deliberation* (mutual communication) between the publics [10]. Deliberative democratic processes recruit a group of participants (a mini public) to do this deliberation. The purpose of deliberation is to expose participants to different ideas, views and opinions [1]. In some cases, participants may change their views after considering the views of their peers. Good deliberation is typically characterised by the deliberants providing reasons or justifications for their views, listening to one another's views and considering one another's views.
Perceived knowledge gains by members	Members have exercised and gained empathy by developing mutual understanding and considering different views and experiences. Members have gained a clearer understanding of each other's opinions. After deliberation, members have a better understanding of both the policy issue and the public decision‐making process in general. The opinion of each member became clearer through deliberation and moved towards informed judgement.	Deliberative democrats consider the informed opinions of publics more normatively valuable than uninformed opinions [15]. If a project can demonstrate that participants became more informed throughout the process, this suggests that the participants' recommendations are less likely to be based on misinformation and self‐interest.
Accessibility and equality of opportunity to speak	All members had equal speaking opportunities, an opportunity to influence the discussions and equal access to any necessary support, tools, or resources during the process. Members had the opportunity to provide ongoing feedback and suggest modifications of the process (such as asking for more time or reporting experienced bias).	In an ideal deliberative democratic process, all participants should have an opportunity to contribute their views and experiences to the discussion, and to have those views deeply considered by their peers [10]. Without opportunities for all participants to contribute, processes risk replicating existing structural inequalities in society and producing recommendations that only reflect the views of certain groups.
Respect and mutual comprehension	Interactions amongst members were respectful. There was careful and active listening, as well as interactive deliberation, that allowed members to weigh each other's views. All members felt heard in the process.	Good deliberation requires respectful communication between jurors [27]. By engaging with one another respectfully, jurors encourage more equal participation and create an environment that allows for free sharing of views and relevant considerations [10]. Demonstrating respect involves listening to one another, challenging one another where appropriate, and mutual comprehension, defined as an attempt to ‘understand the meaning of a speaker's statements to that speaker, rather than viewing the statements as objects to be dismissed, demeaned, manipulated, or destroyed’ [10].
Free decision‐making and response	The implementation of the process was free of interference beyond set roles and processes (i.e., intrusions by experts, steering group members). The final recommendations represent what the members actually think (i.e., members had a final say over the wording of the recommendations). The final decision‐making was non‐coercive, using democratic decision‐making rules (i.e., consensus, majority rule, ranking, etc.). The report fully reflects the judgement of the group, including views that were not supported by the majority. Members were free and supported to contribute a minority report, which appears in the appendix to the main report.	Deliberative democrats describe ideal deliberative processes as having an absence of unequal power or coercive control [10]. Whilst perfect equality is impossible empirically, there are measures that can be undertaken to promote equality amongst participants, and prevent the process from being influenced by external actors in ways that influence the outcome.
Respect for members' privacy	Members' privacy was protected. For more details, see Annex B—Principle 10: Privacy. There was no undesired attention or attempt at interference from the media, stakeholders, or other actors.	Deliberative democratic processes must balance the need for publicity (making the process transparent) with protecting the privacy of the participants. Although publicity is important to ensure processes are accountable to the public, protecting the identities of individual jurors respects their right to privacy, and can prevent corruption through coercion or improper influence [10, 28]

a Text in this column is quoted verbatim from [13].

#### 2.1.2 Framework Items

We used the OECD's [13] criteria to develop a series of items on which Juries should be evaluated. The final framework (File S1) relies on data collected from a participant questionnaire, analysis of documentation and a transcript analysis.

We developed the participant questionnaire (File S2) by collecting existing evaluation questionnaires for deliberative democratic or online qualitative research processes [18, 29, 30, 31, 32, 33, 34] and compiling them into a matrix based on the criteria addressed by each question. As a team, we discussed which questions were most relevant for collecting jurors' views. Where we felt certain criteria were not sufficiently addressed by existing questionnaires, we developed new questions. Because the jury process was already a time‐intensive process for participants, we selected questions sparingly to minimise burden.

The documentation analysis and transcript analysis criteria (File S2) were developed based on the OECD's criteria and descriptions [13]. The final evaluation framework in File S1 triangulates the three sources of information against the OECD's criteria [13]. The evaluation framework provides an outline for how each of the three methods should be evaluated against the OECD's evaluation criteria.

### 2.2 Evaluating a Citizens' Jury on AI in Healthcare

In the final section of this paper, we describe our process for piloting our evaluation tool. The purpose of this section is to describe how the tool can be used in practice, and to demonstrate how the results can be reported transparently and reflexively. First, we provide a brief overview of the AI in Healthcare Jury. Then, we describe our process for evaluating the jury, and finally, our results.

#### 2.2.1 A Citizen's Jury on AI in Healthcare

In March–April 2023, we ran a citizens' jury on the conditions under which publics felt that AI should be used in healthcare. This section provides a brief overview of the process to contextualise the evaluative results below. More detailed information on the jury can be found on the jury website (https://www.uow.edu.au/research/australian-centre-for-health-engagement-evidence-and-values/artificial-intelligence-in-health/), and the full report from the jury is published elsewhere [19].

We conducted a jury to address the question: ‘under what circumstances, if any, should AI be used in Australia's health systems to detect or diagnose disease?’. AI has the potential to improve clinical care, but its implementation may also lead to harm to patients and the healthcare system [35]. In Australia, governance of AI lags behind other countries [36], and there is an identified need for healthcare‐specific strategies for guiding the implementation and oversight of AI technologies [37].

We recruited 30 participants using the independent deliberative democracy recruitment agency, Sortition Foundation (www.sortitionfoundation.org). Six thousand households were randomly selected from the Australia Post database. From those who volunteered to participate, Sortition Foundation used an algorithm to select 31 participants who best matched the Australian population according to gender, age, ancestry, highest level of education and location of residence. Two jurors were unable to attend the face‐to‐face meeting, so the process was completed with 28 jurors.

The jury was conducted over 18 days, with 15 days online and 3 days face‐to‐face. The online component was predominantly conducted using the VisionsLive platform (https://visionslive.com/), which allowed us to host informational videos and provide channels for participants to interact with one another. The face‐to‐face meeting was then held over 3 days in Sydney, where jurors developed recommendations. A full schedule of the Jury is online (https://www.uow.edu.au/research/australian-centre-for-health-engagement-evidence-and-values/artificial-intelligence-in-health/#:~:text=Information%20booklet.-,The%20roadmap,-This%20jury%20process).

#### 2.2.2 The Evaluation Process: Piloting the C‐JuRI Framework

We piloted the C‐JuRI Framework in seven stages.

##### 2.2.2.1 Stage 1—Preparation

**Figure 1:**
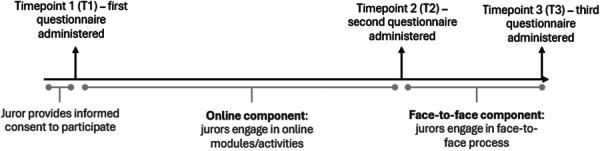
Jury timeline and timepoints at which evaluative questionnaires were administered.

During the jury proceedings, we asked the jurors to complete evaluation questionnaires at three timepoints (Figure 1). The jurors completed the first questionnaire online after providing consent to participate, and the following two questionnaires as paper forms at the beginning and end of the face‐to‐face component, respectively. We assigned each juror a unique number to track their responses between timepoints without identifying individuals by name. We audio‐recorded most sessions during the jury proceedings, including online small group sessions, the jurors' interactions with the experts, sessions where jurors were determining areas for recommendations, and the final session where jurors voted on recommendations.

##### 2.2.2.2 Stage 2—Descriptive Analysis of Survey Data

After the jury proceedings, LC coded the data from the paper questionnaires, and EF combined all timepoints and summarised the results. We recoded the data into dichotomous variables by combining the highest two categories from the Likert‐style scales (e.g., ‘agree’ and ‘strongly agree’) to find the number of jurors who responded positively to each item.

##### 2.2.2.3 Stage 3—Deductive Analysis of Jury Transcripts

We securely transferred the audio recordings of the jury sessions to a professional transcription company. EF read the transcripts and coded quotes using the deductive coding framework (File S2) as a guide.

##### 2.2.2.4 Stage 4—Deductive Analysis of Jury Documentation

We collected documentation from the jury, including all information shown to jurors, meeting minutes from the organising team, planning documentation about the jury objectives, agreements signed by observers and privacy forms signed by jurors. We used the documentation analysis framework (File S2) to extract information from the documentation.

##### 2.2.2.5 Stage 5—Collating Data for Review

Once the survey data were summarised and the textual data were coded, EF began adding the evidence to the relevant sections in the final evaluation framework File S1). We found that some items—particularly those from the jury transcript analysis—had a large amount of evidence. For example, the code ‘jurors provide justifications for their viewpoints’ had many quotes, as transcripts covered over 30 h of audio, and jurors were often sharing views and reasons with one another. Because of the amount of evidence for some items, we found it useful to include only 1–2 pieces of evidence per item, which best demonstrated what the criterion was measuring, rather than including all quotations.

Once the evidence had been copied into the relevant sections of the framework, EF distributed the framework to the evaluation team (S.M.C., Y.S.J.A., A.B.‐M.). The evaluation team were invited to review and evaluate the strength of the evidence.

##### 2.2.2.6 Stage 6—Evaluating the Evidence

Authors E.K.F., S.M.C., Y.S.J.A. and A.B.‐M. met across three 1‐h sessions between March and May 2025 to evaluate the evidence. S.M.C. was the lead moderator of the AI in Healthcare Jury. E.K.F. and Y.S.J.A. had planning and co‐moderating roles. A.B.‐M. is an expert in deliberative democratic methods and was invited to provide an independent view.

In the meetings, we went through each item in the framework and discussed (a) whether there was evidence for the item, and (b) what might constitute stronger evidence. In our discussions, we noted that Juries cannot be ideal, and so we recorded where we had made trade‐offs (e.g., between involving policy experts and maintaining an unbiased framing) and where there was potential to make changes to the method in the future. For each criterion, we recorded ‘strengths’—where the method worked well—and ‘opportunities’—where changes could be made to improve the method.

##### 2.2.2.7 Stage 7—Reporting Results

We tabulated the rating for each individual item and our notes on strengths and opportunities. Results are reported and discussed further below.

#### 2.2.3 Post‐Pilot Amendments to the Framework

After piloting the framework, we identified areas where we felt the framework was not able to sufficiently evaluate the criteria. For example, although the draft framework asked whether there was an established process to prevent interference from observers, there were no items to evaluate whether those processes were followed, which we felt was an important omission. For the transcript analysis and documentation analysis items, we were able to make changes to the framework, re‐collect evidence and re‐evaluate that evidence before presenting results. For the questionnaire, we could not go back and re‐survey the jurors. For simplicity, the framework presented below is the amended post‐pilot framework.

## 3 Results

The fully completed C‐JuRI template is provided in supporting material to this article (File S3). Quantitative summaries of the questionnaire items are provided in File S4. Table 2 shows the ratings item‐by‐item. Of the 77 total items, 67 had evidence to suggest the criteria were met by the AI in healthcare jury. On 11 of those items, we noted opportunities for improvement. Of the remaining 10 items, 3 were not met, 1 was not applicable (as no algorithms were used during the jury), and 6 were questionnaire items that we developed after the pilot as recommendations for future uses of the framework.

**Table 2:** Results of the C‐JuRI framework: criteria, items and whether there was evidence to support each item.

Criterion	Item	Evidence?
Process design		
1.1 Suitable purpose	1.1.1. Did jury documentation show that the jury organisers planned for impact by engaging decision‐makers in the jury	Yes—but opportunities for improvement noted
	1.1.2. Did materials shown to the jurors make it clear how the final jury recommendations will be used	Yes—but opportunities for improvement noted
	1.1.3. Were the objectives of the jury clearly defined during the planning of the jury?	Yes—but opportunities for improvement noted
	1.1.4. Jurors agreed that ‘enough time was provided for each aspect of the process’?	Yes—but opportunities for improvement noted
1.2. Clear and unbiased framing	1.2.1. Jurors agreed that ‘the issue to be discussed was clearly defined’	Yes
	1.2.2. Jurors agreed that ‘the purpose of the event was clear’	Yes
	1.2.3. Jurors agreed that ‘the remit was not biased’	Not included in pilot
1.3. Procedural design involvement	1.3.1 Does the jury documentation contain evidence that policy experts had input into the jury design/remit?	Yes—but opportunities for improvement noted
	1.3.2. Does the jury documentation contain evidence that deliberative democracy experts had input into the jury design/remit?	Yes
	1.3.3. Does the jury documentation contain evidence that subject matter experts had input into the jury design/remit?	Yes
	1.3.4. Is there evidence that any comments from expert stakeholders were recorded by the organisers, with changes made to the jury design where necessary?	Yes
1.4. Transparency and governance	1.4.1. There is evidence that organisers provided ‘ground rules’ to jurors about how they are expected to behave during the process, or gave the jurors the opportunity to develop their own ground rules	Yes
	1.4.2. The jury design was subject to review by an independent ethics committee or another appropriate oversight mechanism (e.g., an expert reference group or governance group)	Yes
	1.4.3. Jurors had all the information necessary to appeal to the ethics committee if they felt that the jury was not meeting its ethical requirements	Yes
	1.4.4. The jury had a public location (e.g., a webpage) where information was available about the process (e.g., objectives, design, governance, funding, participant sampling, outcomes)	Yes
	1.4.5. All parties who had influence over the jury design were reported publicly	No
	1.4.6. Jurors agreed that ‘it was clear how decisions were made in the jury’	Yes
	1.4.7. Jurors agreed that ‘the observers did not interfere with the jury process’	Not included in pilot
1.5. Representativeness and inclusiveness	1.5.1. Participants were selected by civic lottery, or another method which ensures everyone has an equal opportunity to be selected	Yes
	1.5.2. The final group of jurors is broadly demographically representative of the general public (if deemed appropriate, over‐sampling was used to ensure involvement from under‐represented groups)	Yes
	1.5.3. All jurors were remunerated for participating	Yes
	1.5.4. Additional costs were covered to reduce inequitable access to participation (e.g., travel, accommodation, meals, childcare)	Yes
	1.5.5. Jurors agreed that ‘the participants represent a diverse and inclusive sample of publics’	Yes
	1.6.6. Jurors agreed that ‘enough financial assistance was provided for me to be able to engage with the event’	Yes
Deliberative experience		
2.1. Neutrality and inclusivity of facilitation	2.1.1. Documents contain evidence that facilitators were instructed to be neutral	Yes
	2.1.2. Jurors agreed that ‘any conflict that has arisen has been dealt with efficiently by the facilitator’	Yes
	2.1.3. Jurors agreed that ‘all jurors were treated with politeness and respect’	Yes
	2.1.4. Jurors agreed that ‘the facilitators did not try to influence me towards certain recommendations or conclusions’	Not included in pilot
	2.1.5. Transcripts show that facilitators encouraged participation from those who are not used to speaking in public	Yes—but opportunities for improvement noted
	2.1.6. Transcripts show that facilitation used methods to create a safe space for jurors (e.g., helping jurors feel included, that their opinions are valued)	Yes
2.2. Accessible, neutral and transparent use of online tools	2.2.1. Planning materials show that jurors were offered support to access any online tools in the jury (e.g., device loans, internet access, tech support)	Yes
	2.2.2. If algorithms were used in the jury, there is documentation to ensure that they are transparent and auditable (e.g., preference or vote counting, calculations, aggregations)	No algorithms used
	2.2.3. Jurors agreed that ‘enough technological support was provided for the online process’	Yes
	2.2.4. Jurors agreed that ‘accessing the online meeting links was easy’	Yes
	2.2.5. Jurors agreed that ‘I was able to express my ideas during the online process’	Yes
	2.2.6. Jurors agreed that ‘I was able to visualise the other participants easily in the online meetings’	Yes—but opportunities for improvement noted
	2.2.7. Jurors agreed that ‘the online bulletin boards made it easy to communicate with the other jurors’	Yes—but opportunities for improvement noted
	2.2.8. Jurors agreed that ‘the previous online sessions prepared me appropriately to participate in the deliberations’	Yes
2.3. Breadth, diversity, clarity and relevance of the evidence and stakeholders	2.3.1. There is evidence that all jurors were able to access the evidence base	Yes
	2.3.2. The evidence base included evidence in a variety of forms (e.g., video, written)	Yes
	2.3.3. Jurors were made aware, either through written material or during jury sessions, who was responsible for choosing the evidence to be presented	No
	2.3.4. Those involved in choosing the evidence declared any conflicts of interest publicly	No
	2.3.5. Jurors agreed that ‘the information presented was clear and easy to understand’	Yes
	2.3.6. Jurors agreed that ‘the online bulletin boards made it easy to access the evidence packages’	Yes
	2.3.7. Jurors agreed that ‘the expert witnesses were a credible source of information’	Yes
	2.3.8. Jurors agreed that ‘all requested information was provided’	Yes
	2.3.9. Jurors agreed that ‘the evidence packages covered all important information’	Not included in pilot
	2.3.10. Jurors agreed that ‘the expert witnesses represented a broad range of perspectives on the issue’	Yes
2.4. Quality of judgement	2.4.1. Jurors were given resources about how to effectively participate in a deliberative event (e.g., recognising cognitive bias, asking questions to engage with opposing views)	Yes
	2.4.2. Jurors agreed that ‘I felt comfortable with the degree of disagreement during the deliberation’	Yes
	2.4.3. Jurors agreed that ‘I endorsed and adopted points of view that differed from my own’	Yes—but opportunities for improvement noted
	2.4.4. Jurors agreed that ‘I was willing to abide by the group's final decision, even if I personally had a different view’	Yes
	2.4.5. Transcripts show evidence of jurors considering structural issues underlying the policy issue	Yes
	2.4.6. Transcripts show evidence of diversity in jurors’ viewpoints	Yes
	2.4.7. Transcripts show evidence of jurors exposing their assumptions	Yes
	2.4.8. Transcripts show evidence of jurors exploring uncertainties	Yes
	2.4.9. Transcripts show evidence of jurors weighing alternatives and trade‐offs	Yes
	2.4.10. Transcripts show evidence of jurors providing justification for their viewpoints	Yes
	2.4.11. Transcripts show evidence of the jurors engaging with the evidence in their discussion	Yes
2.5. Perceived knowledge gains by members	2.5.1. Jurors agreed that ‘the jury has changed my awareness of different points of view about [POLICY AREA]’	Yes
	2.5.2. Were there any changes between timepoints in the item(s) about jurors’ attitudes towards the policy issue?	Yes
	2.5.3. Were there any changes between timepoints in item ‘how knowledgeable are you about [POLICY ISSUE]’	Yes
2.6. Accessibility and equality of opportunity to speak	2.6.1. Jurors agreed that ‘there were equal opportunities for all jurors to express their views’	Yes—but opportunities for improvement noted
	2.6.2. Jurors agreed that ‘I felt able to suggest changes to the jury process if I felt it was necessary (e.g., requesting more time, or more evidence)’	Not included in pilot
	2.6.3. The transcripts show evidence that modifications were made to the process, where (and if) jurors requested them	Yes
2.7. Respect and mutual comprehension	2.7.1. Jurors agreed that ‘jurors were listening to each other and allowing each other to speak’	Yes
	2.7.2. Jurors agreed that ‘I feel that I was listened to by the facilitator’	Yes
	2.7.3. Jurors agreed that ‘I felt that my opinions were respected by the group’	Yes
	2.7.4. Transcript shows that jurors encouraged one another to share their views	Yes
	2.7.5. Transcript shows that jurors considered one another's views	Yes
2.8. Free decision‐making and response	2.8.1. The final report explains how democratic decision‐making rules (e.g., consensus, majority rule, ranking) were used to generate the final recommendations	Yes
	2.8.2. Documents show that jurors had the opportunity to express alternative views in a minority report.	Yes
	2.8.3. Jurors agreed that ‘it was clear how we were meant to arrive at a decision’	Yes—but opportunities for improvement noted
	2.8.5. Transcripts show that jurors had the final say over the wording of the recommendations	Yes
Respect for members’ privacy	2.9.1. The jury had processes in place to prevent jurors’ identities from being revealed where the juror did not want it to be (e.g., processes preventing photos from being taken unless the juror has given informed consent, anonymisation processes on evaluative surveys)	Yes
	2.9.2. Any observers of the jury were instructed to refrain from interfering in the jury processes	Yes
	2.9.3. Jurors agreed that ‘the jury organisers respected my privacy’	Not included in pilot

Table 3 shows the results of the team's discussion on the strengths and opportunities for each criterion. For almost all criteria, the framework assisted us in identifying both strengths and opportunities for improvement for the jury process.

**Table 3:** Results of the C‐JuRI framework: strengths and opportunities for improvement identified for each criterion.

Criterion	Strengths	Opportunities
1.1. Suitable purpose	The objectives of the jury were clearly defined during the planning stage. Jurors were informed about how their recommendations would be used.	Opportunity for more direct policy influence by engaging decision‐makers who hold relevant policies. Stronger evidence would show that the decision‐makers who held relevant policies were involved in the process and committed to responding to jurors’ recommendations. Time is an opportunity—nearly 1/3 of jurors did not feel that they had enough time. Making the process longer may compromise inclusiveness by increasing the time burden, but this is potentially a sign that the remit could be narrowed to a narrow scope.
1.2. Clear and unbiased framing	Jurors felt that the framing of the jury problem was clear. They felt that the issue to be discussed was clearly defined.	
1.3. Procedural design involvement	Diverse deliberative democracy and subject matter experts were involved in the jury design. Experts provided feedback that was incorporated into the jury design.	Although one policy expert was involved in the expert reference group, there is an opportunity to engage more policymakers (and particularly those who hold relevant policies) in the design of the jury.
1.4. Transparency and governance	We allowed jurors to set their own ground rules, which we returned to throughout the jury process. This was effective. The jury was subject to review by a human research ethics committee, and jurors had all the information necessary to appeal to that committee if they felt the jury was not meeting its requirements. The jury had a webpage where information about the jury was reported publicly.	The names of the members of the expert reference group, who had influence over the design of the jury, were not reported anywhere publicly. This was an oversight.
1.5. Representativeness and inclusiveness	Participants were selected by sortition, which gave every household an equal opportunity of selection. Participants broadly represented the [country's] population. Participants were remunerated $1015 for their time. In addition, they were provided meals, travel and accommodation for the face‐to‐face component.	Opportunity to provide compensation for childcare. This would have improved equity of access for parents.
2.1. Neutrality and inclusivity of facilitation	There was evidence that facilitators supported inclusion and encouraged participation. Jurors felt that they were treated with politeness and respect.	
2.2. Accessible, neutral and transparent use of online tools	The jury had a dedicated staff member to provide tech support and resources to ensure that everyone would be able to participate. The jurors felt that they were able to express their ideas readily during the online process.	Eight of the jurors did not agree that the bulletin boards made it easy to communicate with other jurors—an opportunity to find ways to encourage more interaction with others on the boards.
2.3. Breadth, diversity, clarity and relevance of the evidence and stakeholders	The jurors all found the information accessible and generally agreed that the expert witnesses were credible sources of information.	Opportunity to make jurors aware of who was responsible for choosing the evidence presented. Relatedly, opportunity for those who choose the evidence to publicly declare any conflicts of interest.
2.4. Quality of judgement	Jurors were trained in how to deliberate effectively with their peers. They referred to the evidence in their discussion, provided justification for their viewpoints, and discussed trade‐offs, indicating a high quality of judgement.	
2.5. Perceived knowledge gains by members	Nearly all jurors agreed that participating in the jury changed their awareness of different points of view. The percentage of jurors who felt knowledgeable or very knowledgeable about AI increased from 11% to 86% between T1 and T3, indicating perceived knowledge gains across the group. Several members of the cohort changed their level of support for AI throughout the jury, suggesting that the process changed jurors’ views.	
2.6. Accessibility and equality of opportunity to speak	The jurors exercised their ability to make changes to the jury process when tasks were not working for them.	Whilst 89% of the jurors agreed that there were equal opportunities for all jurors to express their views, three jurors did not feel there were equal opportunities to speak. Qualitative feedback on the evaluation form also suggested that some jurors felt that there were some voices that were louder than others. Opportunity to have co‐facilitators formally monitoring whether any participants are not having a say in small and large group tasks. On the basis of this finding, we also opted to change the questionnaire item to ‘there were opportunities for all jurors to express their views’ to reflect that all jurors should have an opportunity to have their say, but that this will not always necessarily mean that everyone speaks for the same amount of time.
2.7. respect and mutual comprehension	All jurors agreed that they were allowing one another to speak and that their opinions were respected by the group. Transcripts show jurors considering each other's opinions and encouraging one another to share their views. All but one juror felt that they were listened to by the facilitator.	
2.8. Free decision‐making and response	Democratic decision‐making rules were used to draft the final recommendations, with jurors drafting the recommendations in their own words. Most jurors felt that it was clear how they were meant to arrive at a decision.	Two jurors neither agreed nor disagreed to the question ‘it was clear how we were meant to arrive at a decision’. Opportunity for further road mapping of the jury process to explain how the process leads to recommendations.
2.9. Respect for members’ privacy	The jury had processes in place to prevent interference from observers and to ensure participants remained anonymous.	The piloted questionnaire did not contain questions about whether jurors felt that their privacy was respected, so this aspect of the criterion was difficult to evaluate.

## 4 Discussion

We designed the C‐JuRI framework for evaluating quality and rigour in Citizens’ Jury studies. The framework uses documentation analysis, transcript analysis and a juror questionnaire to evaluate 14 criteria based on the OECD's [13] guidance for evaluating deliberative democratic processes. No jury will ever completely fulfil the democratic ideal. The purpose of the framework is not to suggest that an ideal deliberative democratic process is realistic, but to provide practitioners with a practical tool to evaluate the closeness of a process to that ideal.

We piloted the framework on the AI in Healthcare jury (reference redacted). For most of the criteria, there was evidence that items were met. Across the process design criteria, we found that the objectives of the jury were defined early in the planning stage, the framing of the problem was clear and a number of experts were involved in planning the jury. The jury has a publicly available website where information about the jury was published transparently to open the jury process to public commentary [10]. We invited participants by sortition to give each household an equal opportunity of being selected. From those who registered interest, we selected participants to broadly represent the demographics of the public. This ensured that everyone had an equal opportunity of being selected, and that there was representation in the jury from demographic subgroups that are often underrepresented in public involvement initiatives [1], such as the public with only a high school level education, and publics from lower socioeconomic groups. Jurors were also compensated for their travel, meals and time to make the process more accessible.

In the process design criteria, there were three main opportunities for improvement. Firstly, although diverse subject matter experts were involved in the jury, there was limited involvement from relevant policymakers. Involving relevant policymakers would have facilitated more opportunities for impact by ensuring that the remit was relevant to national decision‐making [10]. Secondly, we did not report the names of the expert reference group or their conflicts of interest on the publicly available site. This was an oversight and would have improved the transparency and accountability of the process to the public. Finally, although we calculate that jurors each worked for a minimum of 24 h across 3.5 weeks, nearly 1/3 of jurors felt that they did not have enough time. We had a very broad remit so that jurors could make recommendations that they felt were important, but this may have inadvertently put more time pressure on jurors compared to a more constrained remit.

For the deliberative experience criteria, we found that jurors felt respected by the facilitator and by their peers. All jurors were able to access the evidence packages, and there was substantial evidence of knowledge gains by jurors. There were markers of good deliberation in the transcripts, including jurors referring to the evidence, jurors asking one another to share their views, and jurors weighing up complex trade‐offs. The team had a dedicated staff member for tech support, and jurors were satisfied with most aspects of the online component of the jury. All jurors felt that their opinions were respected by their fellow jurors, which demonstrates effectiveness in designing and moderating the jury in a way that allows for mutual comprehension between jurors with diverse backgrounds and experiences [10].

We noted three main areas for improvement in deliberative experience. Firstly, we did not inform the jurors about how the evidence was chosen. Transparency about how the evidence was selected may have given jurors more opportunity to be involved in shaping the evidence presented to them. Second, eight of the jurors did not agree that the bulletin boards were an effective way of communicating with fellow jurors. This is an opportunity to redesign some of the online components to allow for better asynchronous interactions between jurors. Lastly, there were three jurors who did not feel that all jurors had equal opportunities to speak. In their free‐text feedback, six jurors also commented that some jurors spoke more than others. Although facilitating completely equal speaking opportunities is an unobtainable ideal [10], there is perhaps an opportunity for facilitators to pay closer attention to whether certain jurors are dominating discussions at others’ expense.

Overall, we found the tool insightful for identifying both the strengths of the jury, and opportunities for improvement. The framework identified that the AI in Healthcare Jury was, on balance, effective but not ideal, which is in accordance with how deliberative democrats discuss best practice for empirical deliberative processes [1, 10]. We note that post‐hoc changes to the participant questionnaire meant that our piloting of the tool was incomplete, and that a complete pilot may have resulted in more opportunities for improvement that have not been identified in this analysis.

The framework has some limitations. Firstly, there were aspects of the OECD criteria, such as unbiasedness of the remit and respect for jurors’ privacy, that were not covered in the questionnaire piloted with jurors. In the final framework, we have proposed questions to be asked in future uses of the C‐JuRI framework, which address these omissions, although we have not had the opportunity to pilot these questions. Because we developed the framework after running the AI in Healthcare jury, we did not have an opportunity to review the criteria before running the jury to examine what information we needed to document and collect. For future uses of this framework, we recommend reviewing the framework before running the jury and planning accordingly.

Second, we found it challenging to consistently assess the extent to which each item in the framework was evidenced. For example, although jurors frequently referred to evidence during deliberations, the available data did not allow us to determine whether all jurors engaged with the evidence equally, or whether there were moments where evidence should have been referenced but was not. This made it difficult to assess the degree to which some items were evidenced and to identify opportunities for improvement. As a result, the application of the framework relies on the reflexive judgement of the evaluator, which represents a limitation. Where resources allow, future evaluations could be strengthened by incorporating an independent evaluator to observe deliberative quality in real time. This would support greater transparency and reduce reliance on post‐hoc interpretation of transcripts and documentation.

Third, we were unsure whether evidence of changes in participants’ level of support for AI provided relevant evidence of juror knowledge gains. In the AI in Healthcare jury, we noted an increase in juror support for AI after engaging with the process. However, even if jurors' levels of support for AI had not changed, it would still have been possible that jurors gained knowledge and changed their views with respect to other aspects of the remit. For future uses of this framework, an absence of change in item 2.5.2. may not necessarily indicate that the process did not achieve valuable knowledge gains amongst jurors. The other items in criterion 2.5. may provide better evidence for juror knowledge gains.

Finally, whilst the OECD recommend that samples of jurors be selected to be broadly demographically representative of the broader population, this approach does not necessarily capture all relevant views and experiences in the deliberations. In response, some deliberative democrats have proposed alternative selection methods that purposefully seek discursive diversity among jurors [22]. These measures might include interviewing or surveying potential participants about the policy issue and selecting jurors based on maximising diversity in the views they express. We note, however, that this approach has also been criticised in the deliberative democratic literature. Critics of attempts to recruit for discursive diversity argue that this treats potential participants’ views as if they were stable, privately held attributes that can be measured in advance of deliberation. This assumption is at odds with both deliberative democratic theory and discursive psychology, which emphasise that views are shaped through discourse [38, 39]. Although we do not believe there to be a unilaterally better alternative to seeking demographic representativeness, we note that there may be room for improvement on the framework's criteria in this regard.

## 5 Conclusion

We developed the C‐JuRI framework, a comprehensive framework for assessing the quality of Citizens’ Juries. After piloting the framework on the AI in Healthcare jury, we identified a number of ways in which the jury demonstrated or attempted to demonstrate ideal deliberative democratic practice. We also identified some opportunities for improvements to future processes to better work towards deliberative democratic ideals. As deliberative democratic processes become more commonly used research and policy methods, we anticipate that this framework will be useful for researchers in assessing the quality of their own Citizens’ Juries and policymakers in determining the quality of other Citizens’ Juries.

## Author Contributions

**Emma K. Frost:** conceptualisation, investigation, writing – original draft, methodology, validation, visualisation, formal analysis, data curation. **Yves Saint James Aquino:** investigation, writing – review and editing, methodology, supervision, project administration. **Annette Braunack‐Mayer:** investigation, writhing – review and editing, methodology, supervision. **Lucy Carolan:** investigation, writing – review and editing. **Stacy M Carter:** investigation, writing – review and editing, methodology, supervision, project administration.

## Ethics Statement

The AI in Healthcare Citizens' Jury study was approved by the University of Wollongong Health and Medical Research HREC [2022/314].

## Conflicts of Interest

The authors declare no conflicts of interest.

## Supporting information

SUPPORTING FILE 1 ‐ evaluation framework template.

SUPPORTING FILE 2 ‐ all evaluation items.

SUPPORTING FILE 3 ‐ completed template.

SUPPORTING FILE 4 ‐ quantitative summary.

## Data Availability

The authors have nothing to report.

## Endnotes

1 In the United States, the ‘Citizen Jury’ title is a trademark of the Centre for new Democratic Processes (formerly the Jefferson Centre). The term ‘community jury’ has been used in the literature to refer to adaptations of the Citizens' Jury method.
