# Supplementary material for: Towards Evidence of Rigour in Empirical Deliberative Democratic Methods: Development and Piloting of the C‐JuRI Framework
Source: Health Expect. 2026 Apr 8;29(2):e70608. doi: 10.1111/hex.70608 (PMC13058570; doi:10.1111/hex.70608)
Supplement: Supplementary file 1 — SUPPORTING FILE 1 ‐ evaluation framework template. [file HEX-29-e70608-s004.docx]

C-JuRI evaluation framework template

The following is the template for the C-JuRI evaluation framework. Using the items provided, evaluators should collect evidence from the jury transcripts, documentation, and juror questionnaire. Collected evidence should be pasted into the relevant box in the framework. The leftmost field of the table is pink for the juror survey items, blue for the documentation analysis items, and green for the transcript analysis items.

Once the evidence is collected, the evaluation team should each individually consider the evidence, make any comments on the evidence, and record a rating for each piece of evidence. Then, the team come together and discuss these notes to decide on final ratings and determine strengths of the jury and opportunities for improvement

# Section 1: Process Design

## 1.1: Suitable purpose

|  | Review criteria | Evidence | Rating | Comment |
| --- | --- | --- | --- | --- |
| **D** | 1.1.1. Did jury documentation show that the jury organisers planned for impact by engaging decision-makers in the jury |  | Yes, there is some evidence to suggest that this criterion is met  No, this criterion is not met (either insufficient evidence or evidence does not show that criteria is met) |  |
| **D** | 1.1.2. Did materials shown to the jurors make it clear how the final jury recommendations will be used |  | Yes, there is some evidence to suggest that this criterion is met  No, this criterion is not met (either insufficient evidence or evidence does not show that criteria is met) |  |
| **D** | 1.1.3. Were the objectives of the jury clearly defined during the planning of the jury? |  | Yes, there is some evidence to suggest that this criterion is met  No, this criterion is not met (either insufficient evidence or evidence does not show that criteria is met) |  |
| **Q** | 1.1.4. Jurors agreed that “enough time was provided for each aspect of the process”? |  | Yes, there is some evidence to suggest that this criterion is met  No, this criterion is not met (either insufficient evidence or evidence does not show that criteria is met) |  |

Final reflexive evaluation for **1.1:** **clear and suitable purpose**

| Strengths | Opportunities |
| --- | --- |
|  |  |

## 1.2: Clear and unbiased framing

|  | Review criteria | Evidence | Rating | Comment |
| --- | --- | --- | --- | --- |
| **Q** | 1.2.1. Jurors agreed that “the issue to be discussed was clearly defined” |  | Yes, there is some evidence to suggest that this criterion is met  No, this criterion is not met (either insufficient evidence or evidence does not show that criteria is met) |  |
| **Q** | 1.2.2. Jurors agreed that ‘the purpose of the event was clear’ |  | Yes, there is some evidence to suggest that this criterion is met  No, this criterion is not met (either insufficient evidence or evidence does not show that criteria is met) |  |
| **Q** | 1.2.3. Jurors agreed that ‘the remit was not biased’ |  | Yes, there is some evidence to suggest that this criterion is met  No, this criterion is not met (either insufficient evidence or evidence does not show that criteria is met) |  |

Final reflexive evaluation for **1.2: clear and unbiased framing**

| Strengths | Opportunities |
| --- | --- |
|  |  |

## 1.3: Procedural design involvement

|  | Review criteria | Evidence | Rating | Comment |
| --- | --- | --- | --- | --- |
| **D** | 1.3.1. Does the jury documentation contain evidence that policy experts had input into the jury design/remit? |  | Yes, there is some evidence to suggest that this criterion is met  No, this criterion is not met (either insufficient evidence or evidence does not show that criteria is met) |  |
| **D** | 1.3.2. Does the jury documentation contain evidence that deliberative democracy experts had input into the jury design/remit? |  | Yes, there is some evidence to suggest that this criterion is met  No, this criterion is not met (either insufficient evidence or evidence does not show that criteria is met) |  |
| **D** | 1.3.3. Does the jury documentation contain evidence that subject matter experts had input into the jury design/remit? |  | Yes, there is some evidence to suggest that this criterion is met  No, this criterion is not met (either insufficient evidence or evidence does not show that criteria is met) |  |
| **D** | 1.3.4. Is there evidence that any comments from expert stakeholders were recorded by the organisers, with changes made to the jury design where necessary? |  | Yes, there is some evidence to suggest that this criterion is met  No, this criterion is not met (either insufficient evidence or evidence does not show that criteria is met) |  |

Final reflexive evaluation for **1.3: procedural design involvement.** Based on the evidence above, to what extent did the jury process satisfy this criterion?

| Strengths | Opportunities |
| --- | --- |
|  |  |

## 1.4: Transparency and governance

|  | Review criteria | Evidence | Rating | Comment |
| --- | --- | --- | --- | --- |
| **D** | 1.4.1. There is evidence that organisers provided ‘ground rules’ to jurors about how they are expected to behave during the process, or gave the jurors the opportunity to develop their own ground rules |  | Yes, there is some evidence to suggest that this criterion is met  No, this criterion is not met (either insufficient evidence or evidence does not show that criteria is met) |  |
| **D** | 1.4.2. The jury design was subject to review by an independent ethics committee or another appropriate oversight mechanism (E.g. an expert reference group or governance group) |  | Yes, there is some evidence to suggest that this criterion is met  No, this criterion is not met (either insufficient evidence or evidence does not show that criteria is met) |  |
| **D** | 1.4.3. Jurors had all the information necessary to appeal to the ethics committee if they felt that the jury was not meeting its ethical requirements |  | Yes, there is some evidence to suggest that this criterion is met  No, this criterion is not met (either insufficient evidence or evidence does not show that criteria is met) |  |
| **D** | 1.4.4. The jury had a public location (e.g. a webpage) where information was available about the process (e.g. objectives, design, governance, funding, participant sampling, outcomes) |  | Yes, there is some evidence to suggest that this criterion is met  No, this criterion is not met (either insufficient evidence or evidence does not show that criteria is met) |  |
| **D** | 1.4.5. All parties who had influence over the jury design were reported publicly |  | Yes, there is some evidence to suggest that this criterion is met  No, this criterion is not met (either insufficient evidence or evidence does not show that criteria is met) |  |
| **Q** | 1.4.6. Jurors agreed that “the observers did not interfere with the jury process” |  | Yes, there is some evidence to suggest that this criterion is met  No, this criterion is not met (either insufficient evidence or evidence does not show that criteria is met) |  |

Final reflexive evaluation for **1.4: transparency and governance.** Based on the evidence above, to what extent did the jury process satisfy this criterion?

| Strengths | Opportunities |
| --- | --- |
|  | - Publish ERG online |

## 1.5: representativeness and inclusiveness

|  | Review criteria | Evidence | Rating | Comment |
| --- | --- | --- | --- | --- |
| **D** | 1.5.1. Participants were selected by civic lottery, or another method which ensures everyone has an equal opportunity to be selected |  | Yes, there is some evidence to suggest that this criterion is met  No, this criterion is not met (either insufficient evidence or evidence does not show that criteria is met) |  |
| **D** | 1.5.2. The final group of jurors is broadly demographically representative of the general public (if deemed appropriate, over-sampling was used to ensure involvement from underrepresented groups) |  | Yes, there is some evidence to suggest that this criterion is met  No, this criterion is not met (either insufficient evidence or evidence does not show that criteria is met) |  |
| **D** | 1.5.3. All jurors were renumerated for participating |  | Yes, there is some evidence to suggest that this criterion is met  No, this criterion is not met (either insufficient evidence or evidence does not show that criteria is met) |  |
| **D** | 1.5.4. Additional costs were covered to reduce inequitable access to participation (e.g. travel, accommodation, meals, childcare) |  | Yes, there is some evidence to suggest that this criterion is met  No, this criterion is not met (either insufficient evidence or evidence does not show that criteria is met) |  |
| **Q** | 1.5.5. Jurors agreed that “the participants represent a diverse and inclusive sample of Australians” |  | Yes, there is some evidence to suggest that this criterion is met  No, this criterion is not met (either insufficient evidence or evidence does not show that criteria is met) |  |
| **Q** | 1.5.6. Jurors agreed that “enough financial assistance was provided for me to be able to engage with the event” |  | Yes, there is some evidence to suggest that this criterion is met  No, this criterion is not met (either insufficient evidence or evidence does not show that criteria is met) |  |

Final reflexive evaluation for **1.5: representativeness and inclusiveness.** Based on the evidence above, to what extent did the jury process satisfy this criterion?

| Strengths | Opportunities |
| --- | --- |
|  |  |

# Section 2: Deliberative Experience

## 2.1: Neutrality and inclusivity of facilitation

|  | Review criteria | Evidence | Rating | Comment |
| --- | --- | --- | --- | --- |
| **D** | 2.1.1. Documents contain evidence that facilitators were instructed to be neutral |  | Yes, there is some evidence to suggest that this criterion is met  No, this criterion is not met (either insufficient evidence or evidence does not show that criteria is met) |  |
| **Q** | 2.1.2. Jurors agreed that “any conflict that has arisen has been dealt with efficiently by the facilitator” |  | Yes, there is some evidence to suggest that this criterion is met  No, this criterion is not met (either insufficient evidence or evidence does not show that criteria is met) |  |
| **Q** | 2.1.3. Jurors agreed that “all jurors were treated with politeness and respect” |  | Yes, there is some evidence to suggest that this criterion is met  No, this criterion is not met (either insufficient evidence or evidence does not show that criteria is met) |  |
| **Q** | 2.1.4. Jurors agreed that “the facilitators did not try to influence me towards certain recommendations or conclusions” |  | Yes, there is some evidence to suggest that this criterion is met  No, this criterion is not met (either insufficient evidence or evidence does not show that criteria is met) |  |
| **T** | 2.1.5. Transcripts show that facilitators encouraged participation from those who are not used to speaking in public |  | Yes, there is some evidence to suggest that this criterion is met  No, this criterion is not met (either insufficient evidence or evidence does not show that criteria is met) |  |
| **T** | 2.1.6. Transcripts show that facilitation used methods to create a safe space for jurors (e.g. helping jurors feel included, that their opinions are valued) |  | Yes, there is some evidence to suggest that this criterion is met  No, this criterion is not met (either insufficient evidence or evidence does not show that criteria is met) |  |

Final reflexive evaluation for **2.1 Neutrality and inclusivity of facilitation.** Based on the evidence above, to what extent did the jury process satisfy this criterion?

| Strengths | Opportunities |
| --- | --- |
|  |  |

## 2.2: Accessible, neutral, and transparent use of online tools

|  | Review criteria | Evidence | Rating | Comment |
| --- | --- | --- | --- | --- |
| **D** | 2.2.1. Planning materials show that jurors were offered support to access any online tools in the jury (e.g. device loans, internet access, tech support) |  | Yes, there is some evidence to suggest that this criterion is met  No, this criterion is not met (either insufficient evidence or evidence does not show that criteria is met) |  |
| **D** | 2.2.2. If algorithms were used in the jury, there is documentation to ensure that they are transparent and auditable (e.g. preference or vote counting, calculations, aggregations) |  | Yes, there is some evidence to suggest that this criterion is met  No, this criterion is not met (either insufficient evidence or evidence does not show that criteria is met) |  |
| **Q** | 2.2.3. Jurors agreed that “enough technological support was provided for the online process” |  | Yes, there is some evidence to suggest that this criterion is met  No, this criterion is not met (either insufficient evidence or evidence does not show that criteria is met) |  |
| **Q** | 2.2.4. Jurors agreed that “accessing the online meeting links was easy” |  | Yes, there is some evidence to suggest that this criterion is met  No, this criterion is not met (either insufficient evidence or evidence does not show that criteria is met) |  |
| **Q** | 2.2.5. Jurors agreed that “I was able to express my ideas during the online process” |  | Yes, there is some evidence to suggest that this criterion is met  No, this criterion is not met (either insufficient evidence or evidence does not show that criteria is met) |  |
| **Q** | 2.2.6. Jurors agreed that “I was able to visualise the other participants easily in the online meetings” |  | Yes, there is some evidence to suggest that this criterion is met  No, this criterion is not met (either insufficient evidence or evidence does not show that criteria is met) |  |
| **Q** | 2.2.7. Jurors agreed that “the online bulletin boards made it easy to communicate with the other jurors” |  | Yes, there is some evidence to suggest that this criterion is met  No, this criterion is not met (either insufficient evidence or evidence does not show that criteria is met) |  |
| **Q** | 2.2.8. Jurors agreed that “the previous online sessions prepared me appropriately to participate in the deliberations” |  | Yes, there is some evidence to suggest that this criterion is met  No, this criterion is not met (either insufficient evidence or evidence does not show that criteria is met) |  |

Final reflexive evaluation for **2.2 Accessible, neutral and transparent use of online tools.** Based on the evidence above, to what extent did the jury process satisfy this criterion?

| Strengths | Opportunities |
| --- | --- |
|  |  |

## 2.3. Breadth, diversity, clarity and relevance of the evidence and stakeholders

|  | Review criteria | Evidence | Rating | Comment |
| --- | --- | --- | --- | --- |
| **D** | 2.3.1. There is evidence that all jurors were able to access the evidence base |  | Yes, there is some evidence to suggest that this criterion is met  No, this criterion is not met (either insufficient evidence or evidence does not show that criteria is met) |  |
| **D** | 2.3.2. The evidence base included evidence in a variety of forms (e.g. video, written) |  | Yes, there is some evidence to suggest that this criterion is met  No, this criterion is not met (either insufficient evidence or evidence does not show that criteria is met) |  |
| **D** | 2.3.3. Jurors were made aware, either through written material or during jury sessions, who was responsible for choosing the evidence to be presented |  | Yes, there is some evidence to suggest that this criterion is met  No, this criterion is not met (either insufficient evidence or evidence does not show that criteria is met) |  |
| **D** | 2.3.4. Those involved in choosing the evidence declared any conflicts of interest publicly |  | Yes, there is some evidence to suggest that this criterion is met  No, this criterion is not met (either insufficient evidence or evidence does not show that criteria is met) |  |
| **Q** | 2.3.5. Jurors agreed that “the information presented was clear and easy to understand” |  | Yes, there is some evidence to suggest that this criterion is met  No, this criterion is not met (either insufficient evidence or evidence does not show that criteria is met) |  |
| **Q** | 2.3.6. Jurors agreed that “the online bulletin boards made it easy to access the evidence packages” * |  | Yes, there is some evidence to suggest that this criterion is met  No, this criterion is not met (either insufficient evidence or evidence does not show that criteria is met) |  |
| **Q** | 2.3.7. Jurors agreed that “the expert witnesses were a credible source of information” |  | Yes, there is some evidence to suggest that this criterion is met  No, this criterion is not met (either insufficient evidence or evidence does not show that criteria is met) |  |
| **Q** | 2.3.8. Jurors agreed that “all requested information was provided” |  | Yes, there is some evidence to suggest that this criterion is met  No, this criterion is not met (either insufficient evidence or evidence does not show that criteria is met) |  |
| **Q** | 2.3.9. Jurors agreed that “the evidence packages covered all important information” |  | Yes, there is some evidence to suggest that this criterion is met  No, this criterion is not met (either insufficient evidence or evidence does not show that criteria is met) |  |
| **Q** | 2.3.10. Jurors agreed that “the expert witnesses represented a broad range of perspectives on the issue” |  | Yes, there is some evidence to suggest that this criterion is met  No, this criterion is not met (either insufficient evidence or evidence does not show that criteria is met) |  |

Final reflexive evaluation for **2.3 Breadth, diversity, clarity and relevance of the evidence and stakeholders.** Based on the evidence above, to what extent did the jury process satisfy this criterion?

| Strengths | Opportunities |
| --- | --- |
|  |  |

## 2.4: Quality of judgement

|  | Review criteria | Evidence | Rating | Comment |
| --- | --- | --- | --- | --- |
| **D** | 2.4.1. Jurors were given resources about how to effectively participate in a deliberative event (e.g. recognising cognitive bias, asking questions to engage with opposing views) |  | Yes, there is some evidence to suggest that this criterion is met  No, this criterion is not met (either insufficient evidence or evidence does not show that criteria is met) |  |
| **Q** | 2.4.2. Jurors agreed that “I felt comfortable with the degree of disagreement during the deliberation” |  | Yes, there is some evidence to suggest that this criterion is met  No, this criterion is not met (either insufficient evidence or evidence does not show that criteria is met) |  |
| **Q** | 2.4.3. Jurors agreed that “I endorsed and adopted points of view that differed from my own” |  | Yes, there is some evidence to suggest that this criterion is met  No, this criterion is not met (either insufficient evidence or evidence does not show that criteria is met) |  |
| **Q** | 2.4.4. Jurors agreed that “I was willing to abide by the group’s final decision, even if I personally had a different view” |  | Yes, there is some evidence to suggest that this criterion is met  No, this criterion is not met (either insufficient evidence or evidence does not show that criteria is met) |  |
| **T** | 2.4.5. Transcripts show evidence of jurors considering structural issues underlying the policy issue |  | Yes, there is some evidence to suggest that this criterion is met  No, this criterion is not met (either insufficient evidence or evidence does not show that criteria is met) |  |
| **T** | 2.4.6. Transcripts show evidence of diversity in jurors’ viewpoints |  | Yes, there is some evidence to suggest that this criterion is met  No, this criterion is not met (either insufficient evidence or evidence does not show that criteria is met) |  |
| **T** | 2.4.7. Transcripts show evidence of jurors exposing their assumptions |  | Yes, there is some evidence to suggest that this criterion is met  No, this criterion is not met (either insufficient evidence or evidence does not show that criteria is met) |  |
| **T** | 2.4.8. Transcripts show evidence of jurors exploring uncertainties |  | Yes, there is some evidence to suggest that this criterion is met  No, this criterion is not met (either insufficient evidence or evidence does not show that criteria is met) |  |
| **T** | 2.4.9. Transcripts show evidence of jurors weighing alternatives and trade-offs |  | Yes, there is some evidence to suggest that this criterion is met  No, this criterion is not met (either insufficient evidence or evidence does not show that criteria is met) |  |
| **T** | 2.4.10. Transcripts show evidence of jurors providing justification for their viewpoints |  | Yes, there is some evidence to suggest that this criterion is met  No, this criterion is not met (either insufficient evidence or evidence does not show that criteria is met) |  |
| **T** | 2.4.11. Transcripts show evidence of jurors approaching the process with open-mindedness |  | Yes, there is some evidence to suggest that this criterion is met  No, this criterion is not met (either insufficient evidence or evidence does not show that criteria is met) |  |
| **T** | 2.4.12. Transcripts show evidence of the jurors engaging with the evidence in their discussion |  | Yes, there is some evidence to suggest that this criterion is met  No, this criterion is not met (either insufficient evidence or evidence does not show that criteria is met) |  |

Final reflexive evaluation for **2.4 Quality of judgement.** Based on the evidence above, to what extent did the jury process satisfy this criterion?

| Strengths | Opportunities |
| --- | --- |
|  |  |

## 2.5: Perceived knowledge gains by members

|  | Review criteria | Evidence | Rating | Comment |
| --- | --- | --- | --- | --- |
| **Q** | 2.5.1. Jurors agreed that “the jury has changed my awareness of different points of view about [POLICY AREA]” |  | Yes, there is some evidence to suggest that this criterion is met  No, this criterion is not met (either insufficient evidence or evidence does not show that criteria is met) |  |
| **Q** | 2.5.2. Were there any changes between timepoints in item(s) related to support or opposition to policy issue? |  | Yes, there is some evidence to suggest that this criterion is met  No, this criterion is not met (either insufficient evidence or evidence does not show that criteria is met) |  |
| **Q** | 2.5.3. Were there any changes between timepoints in item “how knowledgeable are you about [POLICY ISSUE]” |  | Yes, there is some evidence to suggest that this criterion is met  No, this criterion is not met (either insufficient evidence or evidence does not show that criteria is met) |  |

Final reflexive evaluation for **2.5 Perceived knowledge gains by members.** Based on the evidence above, to what extent did the jury process satisfy this criterion?

| Strengths | Opportunities |
| --- | --- |
|  |  |

## 2.6: accessibility and equality of opportunity to speak

|  | Review criteria | Evidence | Rating | Comment |
| --- | --- | --- | --- | --- |
| **Q** | 2.6.1. Jurors agreed that “there were opportunities for all jurors to express their views” |  | Yes, there is some evidence to suggest that this criterion is met  No, this criterion is not met (either insufficient evidence or evidence does not show that criteria is met) |  |
| **Q** | 2.6.2. Jurors agreed that “I felt able to suggest changes to the jury process if I felt it was necessary (e.g. requesting more time, or more evidence)” |  | Yes, there is some evidence to suggest that this criterion is met  No, this criterion is not met (either insufficient evidence or evidence does not show that criteria is met) |  |
| **T** | 2.6.3. The transcripts show evidence that modifications were made to the process, where (and if) jurors requested them |  | Yes, there is some evidence to suggest that this criterion is met  No, this criterion is not met (either insufficient evidence or evidence does not show that criteria is met) |  |

Final reflexive evaluation for **2.6 accessibility and equality of opportunity to speak.** Based on the evidence above, to what extent did the jury process satisfy this criterion?

| Strengths | Opportunities |
| --- | --- |
|  |  |

## 2.7: respect and mutual comprehension

|  | Review criteria | Evidence | Rating | Comment |
| --- | --- | --- | --- | --- |
| **Q** | 2.7.1. Jurors agreed that “jurors were listening to each other and allowing each other to speak” |  | Yes, there is some evidence to suggest that this criterion is met  No, this criterion is not met (either insufficient evidence or evidence does not show that criteria is met) |  |
| **Q** | 2.7.2. Jurors agreed that “I feel that I was listened to by the facilitator” |  | Yes, there is some evidence to suggest that this criterion is met  No, this criterion is not met (either insufficient evidence or evidence does not show that criteria is met) |  |
| **Q** | 2.7.3. Jurors agreed that “I felt that my opinions were respected by the group” |  | Yes, there is some evidence to suggest that this criterion is met  No, this criterion is not met (either insufficient evidence or evidence does not show that criteria is met) |  |
| **T** | 2.7.4. Transcript shows that jurors encouraged one another to share their views |  | Yes, there is some evidence to suggest that this criterion is met  No, this criterion is not met (either insufficient evidence or evidence does not show that criteria is met) |  |
| **T** | 2.7.5. Transcript shows that jurors considered one another’s views |  | Yes, there is some evidence to suggest that this criterion is met  No, this criterion is not met (either insufficient evidence or evidence does not show that criteria is met) |  |

Final reflexive evaluation for **2.7 Respect and mutual comprehension.** Based on the evidence above, to what extent did the jury process satisfy this criterion?

| Strengths | Opportunities |
| --- | --- |
|  |  |

## 2.8: Free decision-making and response

|  | Review criteria | Evidence | Rating | Comment |
| --- | --- | --- | --- | --- |
| **D** | 2.8.1. The final report explains how democratic decision-making rules (e.g. consensus, majority rule, ranking) were used to generate the final recommendations |  | Yes, there is some evidence to suggest that this criterion is met  No, this criterion is not met (either insufficient evidence or evidence does not show that criteria is met) |  |
| **D** | 2.8.2. The final report includes a minority report (if appropriate) where jurors had the opportunity to report views that were not shared by the majority |  | Yes, there is some evidence to suggest that this criterion is met  No, this criterion is not met (either insufficient evidence or evidence does not show that criteria is met) |  |
| **Q** | 2.8.3. Jurors agreed that “it was clear how we were meant to arrive at a decision” |  | Yes, there is some evidence to suggest that this criterion is met  No, this criterion is not met (either insufficient evidence or evidence does not show that criteria is met) |  |
| **T** | 2.8.4. Transcripts show that jurors had the final say over the wording of the recommendations |  | Yes, there is some evidence to suggest that this criterion is met  No, this criterion is not met (either insufficient evidence or evidence does not show that criteria is met) |  |

Final reflexive evaluation for **2.8 Free decision-making and response.** Based on the evidence above, to what extent did the jury process satisfy this criterion?

| Strengths | Opportunities |
| --- | --- |
|  |  |

## 2.9: respect for members’ privacy

|  | Review criteria | Evidence | Rating | Comment |
| --- | --- | --- | --- | --- |
| **D** | 2.9.1. The jury had processes in place to prevent jurors' identity from being revealed where the juror did not want it to be (e.g. processes preventing photos being taken unless juror has given informed consent, anonymisation processes on evaluative surveys) |  | Yes, there is some evidence to suggest that this criterion is met  No, this criterion is not met (either insufficient evidence or evidence does not show that criteria is met) |  |
| **D** | 2.9.2. Any observers of the jury were instructed to refrain from interfering in the jury processes |  | Yes, there is some evidence to suggest that this criterion is met  No, this criterion is not met (either insufficient evidence or evidence does not show that criteria is met) |  |
| **Q** | 2.9.3. Jurors agreed that “the jury organisers respected my privacy” |  | Yes, there is some evidence to suggest that this criterion is met  No, this criterion is not met (either insufficient evidence or evidence does not show that criteria is met) |  |

Final reflexive evaluation for **2.9 Respect for members’ privacy.** Based on the evidence above, to what extent did the jury process satisfy this criterion?

| Strengths | Opportunities |
| --- | --- |
|  |  |
