# Supplementary material for: Towards Evidence of Rigour in Empirical Deliberative Democratic Methods: Development and Piloting of the C‐JuRI Framework
Source: Health Expect. 2026 Apr 8;29(2):e70608. doi: 10.1111/hex.70608 (PMC13058570; doi:10.1111/hex.70608)
Supplement: Supplementary file 3 — SUPPORTING FILE 3 ‐ completed template. [file HEX-29-e70608-s001.docx]

C-JuRI evaluation framework template - PILOT

The following is the pilot template for the C-JuRI evaluation framework. Using the items provided, evaluators should collect evidence from the jury transcripts, documentation, and juror questionnaire. Collected evidence should be pasted into the relevant box in the framework. The leftmost field of the table is pink for the juror survey items, blue for the documentation analysis items, and green for the transcript analysis items.

Once the evidence is collected, the evaluation team should each individually consider the evidence, make any comments on the evidence, and record a rating for each piece of evidence. Then, the team come together and discuss these notes to decide on final ratings and determine strengths of the jury and opportunities for improvement.

# Section 1: Process Design

## 1.1: Suitable purpose

|  | Review criteria | Evidence | Rating |  |
| --- | --- | --- | --- | --- |
| **D** | 1.1.1. Did jury documentation show that the jury organisers planned for impact by engaging decision-makers in the jury | From “GUIDE SESSION 1” (information disseminated to jurors in initial plenary)  “*Three Australian Health Research Translation Centres from Victoria, New South Wales and Western Australia respectively, are jointly supporting the community jury. These centres are funded and accredited by the Australian National Health and Medical Research Council to connect health researchers to health systems”* | **Yes, there is some evidence to suggest that this criterion is met**  No, this criterion is not met (either insufficient evidence or evidence does not show that criteria is met) | the information was clear about how impact was planned for, but stronger evidence would show that the decision-makers involved held the policy themselves or were more directly related to the decision. |
| **D** | 1.1.2. Did materials shown to the jurors make it clear how the final jury recommendations will be used | From jury booklet,  *“The recommendations that you make as a group will provide advice to people who design, implement and use artificial intelligence in health systems.”*  From “GUIDE SESSION 1” (information shown to jurors in first plenary)  *“in the end we will have:*  *A list of recommendations from you*  *A list of the reasons for each of those recommendations*  *The* ***recommendations*** *will tell us what areas you think are most important*  *The* ***reasons*** *will tell us why you think they are important*  *The sponsors want to hear what you recommend. They will hear your recommendations on Sunday, and will prepare a response (in due course) which will become public. “* | **Yes, there is some evidence to suggest that this criterion is met**  No, this criterion is not met (either insufficient evidence or evidence does not show that criteria is met) | the information is clear about how the recommendations will be used by the jury team, and that they will be heard by the sponsors, but direct link to policy change is unclear. Separate clarity from suitability of purpose. |
| **D** | 1.1.3. The objectives for the jury were clearly defined during the planning of the jury | From participant booklet,  “This project is an opportunity for members of the community to be directly involved in democratic decision-making. Potential outcomes include providing evidence about community acceptability of healthcare tools that use artificial intelligence, and ways of implementing these tools.  …  In this jury, you will be asked to make recommendations on the following question: Under what circumstances, if any, should artificial intelligence be used in Australian health systems to detect or diagnose disease?” | **Yes, there is some evidence to suggest that this criterion is met**  No, this criterion is not met (either insufficient evidence or evidence does not show that criteria is met) |  |
| **Q** | 1.3.2. Jurors agreed that “enough time was provided for each aspect of the process”? | At both timepoint 2 and timepoint 3, 68% (19/28 jurors) agreed or strongly agreed that enough time was provided for each aspect of the process.  At T2, eight jurors disagreed, and one strongly disagreed. At T3, four neither agreed nor disagreed, four disagreed, and one strongly disagreed. | **Yes, there is some evidence to suggest that this criterion is met**  No, this criterion is not met (either insufficient evidence or evidence does not show that criteria is met) |  |

Final reflexive evaluation for **1.1:** **clear and suitable purpose**

| Strengths | Opportunities |
| --- | --- |
| - Information presented to jurors was clear | - Opportunity for more direct policy influence by engaging decision-makers who hold policies themselves - Time is an opportunity – jurors felt that they did not have enough time – but more time would have compromised inclusiveness because larger time commitments would prevent people from engaging with the project. Potentially an opportunity to rescope the question to something narrower, although this would change the relevance of the remit and give jurors less freedom to make recommendations that they felt were important. - Jurors had to redraft by hand, might have felt like they were working iteratively and not moving forward |

## 1.2: Clear and unbiased framing

|  | Review criteria | Evidence | Rating |  |
| --- | --- | --- | --- | --- |
| **Q** | 1.2.1. Jurors agreed that “the issue to be discussed was clearly defined” | 96% (27/28 jurors) agreed or strongly agreed that ‘the issue to be discussed was clearly defined’. One juror selected ‘neither agree nor disagree’ | **Yes, there is some evidence to suggest that this criterion is met**  No, this criterion is not met (either insufficient evidence or evidence does not show that criteria is met) |  |
| **Q** | 1.2.2. Jurors agreed that ‘the purpose of the event was clear’ | 93% (26/28 jurors) agreed or strongly agreed that ‘the purpose of the event was clear’. Two jurors selected ‘neither agree nor disagree’. | **Yes, there is some evidence to suggest that this criterion is met**  No, this criterion is not met (either insufficient evidence or evidence does not show that criteria is met) |  |

Final reflexive evaluation for **1.2: clear and unbiased framing**

| Strengths | Opportunities |
| --- | --- |
| - Clarity of framing - Remit was unbiased – very broad, gave jurors plenty of opportunities to make the recommendations that they saw fit. |  |

## 1.3: Procedural design involvement

|  | Review criteria | Evidence | Rating |  |
| --- | --- | --- | --- | --- |
| **D** | 1.4.1. Does the jury documentation contain evidence that policy experts had input into the jury design/remit? | Policy expert from Dept. Health and Aged Care was involved in the expert reference group. Made suggestions for jury case studies in expert reference group meeting on 23 November 2022. From ERG minutes:  [GOVERNMENT DEPT HEALTH ADVISOR]: if we’re building examples around the questions, trying to use examples of where AI is already in use e.g. clinical situations where it is in use. Clinical professions especially in radiology so might be useful to build examples around radiology | **Yes, there is some evidence to suggest that this criterion is met**  No, this criterion is not met (either insufficient evidence or evidence does not show that criteria is met) |  |
| **D** | 1.4.2. Does the jury documentation contain evidence that deliberative democracy experts had input into the jury design/remit? | Two investigators are deliberarive democracy experts, and had oversight over the jury design. From TAWSYN_ERG_Minutes_23Nov2022:  • [health consumer advocate] suggested that it might be helpful to have an expert on consumer rights.  • [HTA expert] agreed with Jen, adding that there is an assumption among consumers that when a technology is approved that it is absolutely safe and effective.  • [DD expert 1] agreed that harms and safety should be covered in topics 5 and 6  • [AI expert] asked about participants having different levels of education. AI is a relatively complicated topic, and issues tend to be subtle and technical. [AI expert] asked whether there’s room for participant self-evaluation of their awareness of AI to enhance study analysis. He suggested a debate-like session with 3 experts discussing one topic, and asking participants which expert they agree with.  • [DD expert 2] stated that the debate format was a good suggestion, but there is a need to ensure the presentations are balanced and that the scope covered by experts are clear. | **Yes, there is some evidence to suggest that this criterion is met**  No, this criterion is not met (either insufficient evidence or evidence does not show that criteria is met) |  |
| **D** | 1.4.3. Does the jury documentation contain evidence that subject matter experts had input into the jury design/remit? | See evidence above. | **Yes, there is some evidence to suggest that this criterion is met**  No, this criterion is not met (either insufficient evidence or evidence does not show that criteria is met) |  |
| **D** | 1.4.4. Is there evidence that any comments from expert stakeholders were recorded by the organisers, with changes made to the jury design where necessary? | The note “FB FROM THE ERG ABOUT THE JURY” summarises the feedback from the expert reference group for consideration in the jury design  “[ERG suggested] Examples that are easy to understand and easily accessible – something about diagnosis and imaging would be good – radiology seems to be the area where it’s most used –… CT scan that would be used in lung cancer screening … looking at a 3D nodule over time – it has to be looked at not just in 2D but in 3D – Ai is able to determine volume over time in a way that you can’t do without AI – probably will work as a second reader with the radiologist …“  This example was incorporated into information booklet for jurors:  “Sybil™ is an Artificial Intelligence algorithm designed to analyse images from CT scans. Sybil™ works without help from a radiologist. Sybil™ predicts the risk of a patient developing lung cancer within six years.” | **Yes, there is some evidence to suggest that this criterion is met**  No, this criterion is not met (either insufficient evidence or evidence does not show that criteria is met) |  |

Reflexive prompts:

- To what extent, on reflection, were a diverse range of experts able to have input into the jury design?
- How was the involvement of experts balanced with the need to ensure that the framing of the jury was clear and unbiased?

Final reflexive evaluation for **1.4: procedural design involvement.** Based on the evidence above, to what extent did the jury process satisfy this criterion?

| Strengths | Opportunities |
| --- | --- |
| - Clarity and unbiasedness was the priority. Involvement of experts was a second priority. For better or for worse. | - We had more content experts than policy experts - Experts were diverse, but mostly not policy experts. |

## 1.4: Transparency and governance

|  | Review criteria | Evidence | Rating |  |
| --- | --- | --- | --- | --- |
| **D** | 1.5.1. There is evidence that organisers provided ‘ground rules’ to jurors about how they are expected to behave during the process, or gave the jurors the opportunity to develop their own ground rules | From “GUIDE SESSION 1” (Runsheet for initial plenary, this was part of the main facilitator’s script)  *“****5:55 Ground rules – 20 minutes***  *Ground rules are really important. We’re going to talk about them now, and then again later when we meet face to face.*  *Ground rules are there to make sure that this is a fun and safe experience for everyone.*  ***[LAUNCH DOCUMENT & share screen – make sure your guide isn’t visible]***  *…*  *I’m going to ask you to raise your hand and suggest ground rules that you think we should follow for this process.*  *If you’re not keen to speak, you can put suggestions into the chat.*  *What rules should we follow while we’re working together?*  ***Scribe or cut and paste their suggestions into the document***  *Live summarise their ideas from the chat into the document – get some agreement on what the issues are*  *“*  The jurors developed ground rules that are recorded in ‘ground rules for the community jury.pdf’  “1. Show respect and create a supportive environment - everyone is equal  2. Everyone’s contribution matters  3. Commit to the process and the schedule, play an active role” | **Yes, there is some evidence to suggest that this criterion is met**  No, this criterion is not met (either insufficient evidence or evidence does not show that criteria is met) |  |
| **D** | 1.5.2. The jury design was subject to review by an independent ethics committee or another appropriate oversight mechanism (E.g. an expert reference group or governance group) | From jury manuscript:  “This project was approved by the xx Human Research Ethics Committee  (xx).”  In addition, the jury design was reviewed by an expert reference group comprised of various subject matter experts in healthcare and AI. From TAWSYN_ERG_AGENDA_23Nov2022_Final, items are  5. “community jury plans”  6. “feedback/questions”  See evidence for 1.4.2. and 1.4.4. for feedback received through this ERG process. | **Yes, there is some evidence to suggest that this criterion is met**  No, this criterion is not met (either insufficient evidence or evidence does not show that criteria is met) |  |
| **D** | 1.5.3. Jurors had all the information necessary to appeal to the ethics committee if they felt that the jury was not meeting its ethical requirements | From the participant information statement that jurors read before giving consent to participate:  *“(14) What if I have a complaint or any concerns about the study?*  *This study has been reviewed by the Medical Human Research Ethics Committee at the University of Wollongong (Reference: 2022/314). In the event of any concerns/complaints regarding how the research is conducted, please contact the UOW Ethics Officer, by phone on +61 2 4239 2191 or email via uow humanethics@uow.edu.au).”* | **Yes, there is some evidence to suggest that this criterion is met**  No, this criterion is not met (either insufficient evidence or evidence does not show that criteria is met) |  |
| **D** | 1.5.4. The jury had a public location (e.g. a webpage) where information was available about the process (e.g. objectives, design, governance, funding, participant sampling, outcomes) | Information about the process, including the information booklet for participants, the remit, the expert witnesses, and the study team is online at this location:  <https://www.uow.edu.au/the-arts-social-sciences-humanities/research/acheev/artificial-intelligence-in-health/> | **Yes, there is some evidence to suggest that this criterion is met**  No, this criterion is not met (either insufficient evidence or evidence does not show that criteria is met) |  |
| **D** | 1.5.5. All parties who had influence over the jury design were reported publicly | The names of the experts and research team were published publicly at the link in 1.5.4. **we did not publish the names of the ERG members? | Yes, there is some evidence to suggest that this criterion is met  **No, this criterion is not met (either insufficient evidence or evidence does not show that criteria is met)** | Opportunity for next jury to publish this. Oversight for AI jury. |
| **Q** | 1.5.6. Jurors agreed that “it was clear how decisions were made in the jury” | 93% (26/28 jurors) agreed or strongly agreed that “it was clear how decisions were made in the jury”. One juror neither agreed or disagreed, and one disagreed. | **Yes, there is some evidence to suggest that this criterion is met**  No, this criterion is not met (either insufficient evidence or evidence does not show that criteria is met) |  |

Final reflexive evaluation for **1.5: transparency and governance.** Based on the evidence above, to what extent did the jury process satisfy this criterion?

| Strengths | Opportunities |
| --- | --- |
| - Allowing jurors to decide on their own ground rules was an effective approach - Observers signed an agreement which prevented any observers in the jury from influencing the process - The jury design was subject to review from a HREC - There was no interference from the media during the process | - Publish membership of the ERG online - Publicise all parties that had influence over the remit and information presented. |

## 1.5: representativeness and inclusiveness

|  | Review criteria | Evidence | Rating |  |
| --- | --- | --- | --- | --- |
| **D** | 1.6.1. Participants were selected by civic lottery, or another method which ensures everyone has an equal opportunity to be selected | From the jury report (p410)  “The independent, not-for-profit, deliberative democracy recruitment agency, Sortition Foundation (https://www.sortitionfoundation.org), recruited thirty Australian residents for this jury. To ensure that each Australian resident had an equal chance of being invited, Sortition Foundation mailed invitations to 6000 households randomly selected from the Australia Post database in February 2023.” | **Yes, there is some evidence to suggest that this criterion is met**  No, this criterion is not met (either insufficient evidence or evidence does not show that criteria is met) |  |
| **D** | 1.6.2. The final group of jurors is broadly demographically representative of the general public (if deemed appropriate, over-sampling was used to ensure involvement from underrepresented groups) | From the jury report (p410)  “From 109 unique eligible respondents (response rate, 1.8%), Sortition Foundation used an algorithm for the stratified random selection of 31 participants according to gender, age, ancestry, highest level of education, location of residence…”  From the jury report (p412)  “The demographic characteristics of the jury were similar to those of the Australian population (Box 2).”  Link to the paper to look at Box 2: <https://onlinelibrary.wiley.com/doi/pdfdirect/10.5694/mja2.52283#page=7.93> | **Yes, there is some evidence to suggest that this criterion is met**  No, this criterion is not met (either insufficient evidence or evidence does not show that criteria is met) |  |
| **D** | 1.6.3. All jurors were renumerated for participating | From the jury report (p410)  “Each juror received $1015 as compensation for their participation…” | **Yes, there is some evidence to suggest that this criterion is met**  No, this criterion is not met (either insufficient evidence or evidence does not show that criteria is met) |  |
| **D** | 1.6.4. Additional costs were covered to reduce inequitable access to participation (e.g. travel, accommodation, meals, childcare) | From the jury report (p410)  “…we booked and paid for travel, accommodation, and all meals for the face-to-face meeting” | **Yes, there is some evidence to suggest that this criterion is met**  No, this criterion is not met (either insufficient evidence or evidence does not show that criteria is met) |  |
| **Q** | 1.6.5. Jurors agreed that “the participants represent a diverse and inclusive sample of Australians” | 89% (25/28) of the jurors agreed or strongly agreed (T3). Three neither agreed or disagreed. | **Yes, there is some evidence to suggest that this criterion is met**  No, this criterion is not met (either insufficient evidence or evidence does not show that criteria is met) |  |
| **Q** | 1.6.6. Jurors agreed that “enough financial assistance was provided for me to be able to engage with the event” | 96% (27/28) of the jurors agreed or strongly agreed (T3). One disagreed. | **Yes, there is some evidence to suggest that this criterion is met**  No, this criterion is not met (either insufficient evidence or evidence does not show that criteria is met) |  |

Final reflexive evaluation for **1.6: representativeness and inclusiveness.** Based on the evidence above, to what extent did the jury process satisfy this criterion?

| Strengths | Opportunities |
| --- | --- |
|  |  |

# Section 2: Deliberative Experience

## 2.1: Neutrality and inclusivity of facilitation

|  | Review criteria | Evidence | Rating |  |
| --- | --- | --- | --- | --- |
| **D** | 2.1.1. Documents contain evidence that facilitators were instructed to be neutral | From “Overall session guide – TAWSYN”. Instructions for small group moderators  “You are there just to listen, encourage where needed, remind the group to record insights, and be ready to pass insights on to the next group.  It’s very important that you remain neutral – don’t extol or condemn the content of what jurors say – but do step in if they aren’t respecting the ground rules, or if you need to direct them back to the task “ | **Yes, there is some evidence to suggest that this criterion is met**  No, this criterion is not met (either insufficient evidence or evidence does not show that criteria is met) |  |
| **Q** | 2.1.2. Jurors agreed that “any conflict that has arisen has been dealt with efficiently by the facilitator” | At T2, 59% (N=16/27) agreed or strongly agreed that ‘any conflict that has arisen has been dealt with efficiently by the facilitator’. This increased to 96% (N=27/28) by the end of the jury.  Note: the online component of the jury had very few moments of conflict. Most conflict happened during face-to-face component. | **Yes, there is some evidence to suggest that this criterion is met**  No, this criterion is not met (either insufficient evidence or evidence does not show that criteria is met) |  |
| **Q** | 2.1.3. Jurors agreed that “all jurors were treated with politeness and respect” | At T2, 100% (N=28) of jurors agreed or strongly agreed that ‘all jurors were treated with politeness and respect’. At T3, 96% of jurors agreed with this statement (N=27/28). | **Yes, there is some evidence to suggest that this criterion is met**  No, this criterion is not met (either insufficient evidence or evidence does not show that criteria is met) |  |
| **T** | 2.1.4. Transcripts show that facilitators encouraged participation from those who are not used to speaking in public | From Session 7a:  “Thank you, {NAME REMOVED}.  We have a few more minutes left on this task.  Is it okay, um, if – uh, who haven't we heard from {NAME REMOVED}, {NAME REMOVED} and {NAME REMOVED} to go next, please?  And {NAME REMOVED}.  And {NAME REMOVED}.”  From Session 7b:  “And what would be great actually, if we could have a couple of people that have never spoken in this big group before.  Um, could I have a couple of people just to give us some reflections on the discussions that they've been a part of today? Um, anything that really stands out for you that you are going to take into the conversations on the weekend in [city where jury was held]?  I have a couple of people maybe just to, um, maybe you could just use the reaction button at the bottom of the screen to raise your hand.  Um, if you haven't spoken in this big group before, give us a reflection. “ | **Yes, there is some evidence to suggest that this criterion is met**  No, this criterion is not met (either insufficient evidence or evidence does not show that criteria is met) |  |
| **T** | 2.1.5. Transcripts show that facilitation used methods to create a safe space for jurors | From Session 7b. Context: one juror suggested that other jurors lack technical knowledge of AI. The juror with IT experience offered to teach other jurors about technical aspects of AI.  “Thanks {NAME REMOVED}.  Um, and I think it's really important to emphasise actually these processes only work because we have a really diverse range of people involved.  You know, that diversity is actually so critical to the method 'cause this - this instance that we're talking about now and bringing AI into healthcare systems, it's gonna affect everyone.  And so this conversation has to include people like {NAME REMOVED} that has quite high level technical skills, and people who actually have not really, ah, engaged in a technical way with IT systems at all, and - and we all - the thing that we all have in common is that at some point we all end up in healthcare.  You know, all of us actually engage with the health system at some point.  Um, that's relevant to all of us.  So thanks {NAME REMOVED} for the generous offer of providing some technical information.  I know some people are still feeling a bit overwhelmed by information and that's okay.  That - that's quite common at this point.  Um, it's really kind of jurors that have more IT background to maybe, um, offer to provide some explanations or some support, and we are really happy to do that as well.  But don't feel like you've gotta understand the tech to be able to contribute, um, because you'll have experience that will be critical to the conversation to contribute.” | **Yes, there is some evidence to suggest that this criterion is met**  No, this criterion is not met (either insufficient evidence or evidence does not show that criteria is met) |  |

Final reflexive evaluation for **2.1 Neutrality and inclusivity of facilitation.** Based on the evidence above, to what extent did the jury process satisfy this criterion?

| Strengths | Opportunities |
| --- | --- |
| - Evidence of attempts to support inclusion – also evidence that jurors felt included | - Live documentation of this would be better to evaluate holistically whether facilitation was good |

## 2.2: Accessible, neutral, and transparent use of online tools

|  | Review criteria | Evidence | Rating |  |
| --- | --- | --- | --- | --- |
| **D** | 2.2.1. Planning materials show that jurors were offered support to access any online tools in the jury (e.g. device loans, internet access, tech support) | The team had a dedicated employee for providing assistance to jurors who needed support with technology. Jurors were provided with tablets and prepaid SIM cards if they required them. In ‘agenda and minutes 060223’, the team plan logistics for sending tablets with prepaid Telstra SIM cards to jurors.  “Samsung tablets … how to post to participants? … TNT couriers $82 2-3 days to Kalgoorlie WA … Put in SIM cards … Zoom downloaded, Visions live icon saved … Set up ZOOM account for the participants with the details they supply” | **Yes, there is some evidence to suggest that this criterion is met**  No, this criterion is not met (either insufficient evidence or evidence does not show that criteria is met) | RA also did additional coaching and support where jurors required it. |
| **D** | 2.2.2. If algorithms were used in the jury, there is documentation to ensure that they are transparent and auditable (e.g. preference or vote counting, calculations, aggregations) | No algorithms were used in the jury. | N/A |  |
| **Q** | 2.2.3. Jurors agreed that “enough technological support was provided for the online process” | 89% (N=25/28) jurors agreed that ‘enough technological support was provided for the online process’ | **Yes, there is some evidence to suggest that this criterion is met**  No, this criterion is not met (either insufficient evidence or evidence does not show that criteria is met) |  |
| **Q** | 2.2.4. Jurors agreed that “accessing the online meeting links was easy” | 96% (N=27/28) jurors agreed that ‘accessing the online meeting links was easy’ | **Yes, there is some evidence to suggest that this criterion is met**  No, this criterion is not met (either insufficient evidence or evidence does not show that criteria is met) |  |
| **Q** | 2.2.5. Jurors agreed that “I was able to express my ideas during the online process” | 93% of jurors (26/28) agreed or strongly agreed that “I was able to readily express my ideas during the online process” | **Yes, there is some evidence to suggest that this criterion is met**  No, this criterion is not met (either insufficient evidence or evidence does not show that criteria is met) |  |
| **Q** | 2.2.6. Jurors agreed that “I was able to visualise the other participants easily in the online meetings” | 82% of jurors (23/28) agreed or strongly agreed that “I was able to visualise the other participants easily in the Zoom meetings” | **Yes, there is some evidence to suggest that this criterion is met**  No, this criterion is not met (either insufficient evidence or evidence does not show that criteria is met) |  |
| **Q** | 2.2.7. Jurors agreed that “the online bulletin boards made it easy to communicate with the other jurors”* | 71% (N=20/28) of jurors agreed that “the online bulletin boards made it easy to communicate with the other jurors”* | **Yes, there is some evidence to suggest that this criterion is met**  No, this criterion is not met (either insufficient evidence or evidence does not show that criteria is met) | Potential opportunity to increase opportunities for jurors to communicate thru bulletin boards. |
| **Q** | 2.2.8. Jurors agreed that “the previous online sessions prepared me appropriately to participate in the deliberations” | 89% (25/28) of jurors agreed that “the previous online sessions prepared me appropriately to participate in the deliberations” | **Yes, there is some evidence to suggest that this criterion is met**  No, this criterion is not met (either insufficient evidence or evidence does not show that criteria is met) |  |

*jury processes that do not employ online bulletin boards may wish to change or retire this question

Final reflexive evaluation for **2.2 Accessible, neutral and transparent use of online tools.** Based on the evidence above, to what extent did the jury process satisfy this criterion?

| Strengths | Opportunities |
| --- | --- |
| - Dedicated staff for tech support - Dedicated resources for tech support - Jurors felt that they were able to express their ideas during the online process. – had an online component that was effective | - Jurors were not as satisfied with the bulletin boards – find new ways of getting them to interact with bulletin boards? |

## 2.3. Breadth, diversity, clarity and relevance of the evidence and stakeholders

|  | Review criteria | Evidence | Rating |  |
| --- | --- | --- | --- | --- |
| **D** | 2.3.1. There is evidence that all jurors were able to access the evidence base | VisionsLive reporting confirmed that all jurors accessed the evidence online and watched the expert video presentations. | **Yes, there is some evidence to suggest that this criterion is met**  No, this criterion is not met (either insufficient evidence or evidence does not show that criteria is met) |  |
| **D** | 2.3.2. The evidence base included evidence in a variety of forms (e.g. video, written) | The evidence base included videos (from experts, case study videos), and written materials (transcripts of expert Q&A sessions, additional evidence summaries). | **Yes, there is some evidence to suggest that this criterion is met**  No, this criterion is not met (either insufficient evidence or evidence does not show that criteria is met) |  |
| **D** | 2.3.3. Jurors were made aware, either through written material or during jury sessions, who was responsible for choosing the evidence to be presented |  | Yes, there is some evidence to suggest that this criterion is met  **No, this criterion is not met (either insufficient evidence or evidence does not show that criteria is met)** | Opportunity to make reasons for evidence selection clear in future juries. |
| **D** | 2.3.4. Those involved in choosing the evidence declared any conflicts of interest publicly |  | Yes, there is some evidence to suggest that this criterion is met  **No, this criterion is not met (either insufficient evidence or evidence does not show that criteria is met)** | Opportunity to make parties involved in selecting the evidence clear to the jurors. |
| **Q** | 2.3.5. Jurors agreed that “the information presented was clear and easy to understand” | 89% (25/28) agreed that “the information presented was clear and easy to understand” | **Yes, there is some evidence to suggest that this criterion is met**  No, this criterion is not met (either insufficient evidence or evidence does not show that criteria is met) |  |
| **Q** | 2.3.6. Jurors agreed that “the online bulletin boards made it easy to access the evidence packages” * | 89% (25/28) agreed that “the online bulletin boards made it easy to access the evidence packages” | **Yes, there is some evidence to suggest that this criterion is met**  No, this criterion is not met (either insufficient evidence or evidence does not show that criteria is met) |  |
| **Q** | 2.3.7. Jurors agreed that “the expert witnesses were a credible source of information” | 88% (23/26) agreed that “the expert witnesses were a credible source of information” | **Yes, there is some evidence to suggest that this criterion is met**  No, this criterion is not met (either insufficient evidence or evidence does not show that criteria is met) |  |
| **Q** | 2.3.8. Jurors agreed that “all requested information was provided” | At T2, 93% (26/28) agreed that “all requested information was provided”. This increased to 100% (28/28) at T3. | **Yes, there is some evidence to suggest that this criterion is met**  No, this criterion is not met (either insufficient evidence or evidence does not show that criteria is met) |  |
| **Q** | 2.3.9. Jurors agreed that “the evidence packages covered all important information” | At T2, 44% (12/27) jurors DISAGREED that there were important issues that the evidence packages DID NOT cover (i.e., they agreed that the evidence packages covered all important issues). At T3, only 36% of jurors disagreed with this question. | Yes, there is some evidence to suggest that this criterion is met  No, this criterion is not met (either insufficient evidence or evidence does not show that criteria is met) | Uncertain – negative framing makes this difficult to interpret – changed the negative framing of this question in the final version of the questionnaire |
| **Q** | 2.3.10. Jurors agreed that “the expert witnesses represented a broad range of perspectives on the issue” | At T2, 85% (22/26) jurors agreed that “the expert witnesses represented a broad range of perspectives on the issue”. This increased to 89% (25/28) at the conclusion of the jury. | **Yes, there is some evidence to suggest that this criterion is met**  No, this criterion is not met (either insufficient evidence or evidence does not show that criteria is met) |  |

*Jury processes that do not use bulletin boards may wish to alter this question (e.g. “it was easy to access to evidence packages”)

Final reflexive evaluation for **2.3 Breadth, diversity, clarity and relevance of the evidence and stakeholders.** Based on the evidence above, to what extent did the jury process satisfy this criterion?

| Strengths | Opportunities |
| --- | --- |
| - Jurors found evidence accessible and comprehensive | - Report to the jurors how evidence was chosen - Report conflicts of interest of those people |

## 2.4: Quality of judgement

|  | Review criteria | Evidence | Rating |  |
| --- | --- | --- | --- | --- |
| **D** | 2.4.1. Jurors were given resources about how to effectively participate in a deliberative event (e.g. recognising cognitive bias, asking questions to engage with opposing views) | Early online sessions from the jury focussed on developing deliberative skills and critical thinking skills. From “Overall session guide”   1. Plenary critical skills task – watch video about cognitive bias, say how it’s relevant 2. Moving between pairs and plenary – what seems important and why – understanding the others’ perspective + listening   Social styles activity. | **Yes, there is some evidence to suggest that this criterion is met**  No, this criterion is not met (either insufficient evidence or evidence does not show that criteria is met) |  |
| **Q** | 2.4.2. Jurors agreed that “I felt comfortable with the degree of disagreement during the deliberation” | 89% (25/28) jurors agreed that “I felt comfortable with the degree of disagreement during the deliberation” | **Yes, there is some evidence to suggest that this criterion is met**  No, this criterion is not met (either insufficient evidence or evidence does not show that criteria is met) |  |
| **Q** | 2.4.3. Jurors agreed that “I endorsed and adopted points of view that differed from my own” | 79% (22/28) agreed that “I endorsed and adopted points of view that differed from my own” | **Yes, there is some evidence to suggest that this criterion is met**  No, this criterion is not met (either insufficient evidence or evidence does not show that criteria is met) | Potential opportunity to encourage adopting different points of view throughout the jury. |
| **Q** | 2.4.4. Jurors agreed that “I was willing to abide by the group’s final decision, even if I personally had a different view” | 93% (26/28) agreed that “I was willing to abide by the group’s final decision, even if I personally had a different view” | **Yes, there is some evidence to suggest that this criterion is met**  No, this criterion is not met (either insufficient evidence or evidence does not show that criteria is met) |  |
| **T** | 2.4.5. Transcripts show evidence of jurors considering structural issues underlying the policy issue | In session 14, jurors considered how AI could lead to an increase in inequity of access to care for people from lower socioeconomic backgrounds. They formulated their recommendation to consider equity.  *“P3:                   So the goal is, um, fairness?*  *P1:                   Not just the Jones family ...  Not just - someone can afford it and someone not - - -*  *P2:                   Dictated by socioeconomic.  So really, yeah, so not dictated by socioeconomic groups.*  *P1:                   Yes.  Not just like people could afford to access.*  *P3:                   Yeah.  So we happy with equitable health outcomes.  Yes.  Okay, and then I'm gonna say equitable health - - -*  *P1:                   Health outcomes.*  *…*  *P3:                   Can we, even broaden that, um, groups because, um, so we've got, again, a really - so people with disability, LGBTIQ, First nations.  Um, so how do we - - -* | **Yes, there is some evidence to suggest that this criterion is met**  No, this criterion is not met (either insufficient evidence or evidence does not show that criteria is met) | Also, many of the final recommendations were related to equity and governane. |
| **T** | 2.4.6. Transcripts show evidence of diversity in jurors’ viewpoints | In Session 7b (online), jurors shared differing views on whether they felt AI would benefit healthcare.  *P6:         … a couple of other people have said virtually the same thing.  [using AI is] like taking the humanity out of it.  A lot of people that present to doctors - an enormous amount of people that present to doctors, um, the - the majority of their problems are psychological problems.  Um, and I know there's an AI out there that can tell if you're depressed or whatever, but that's - I mean, it just manifests into physical problems.  A human being, doctor worth their wage should be able to pick that up.  And - and AI can say, well, if you keep doing the same thing you're doing now, this is what's gonna happen.  But that patient could walk out of that doctor's office that day and totally change their life around.  The humanity aspect just can't be taken away.  I think it's great for screening and everything, like, the cost, well, we don't know, we don't have that information.  But you just can't take humanity out of the medical system …*  *P3:         {NAME REMOVED}, um, that's in regards to what you're saying, I, um, feel as though, ah, with the AI being used for screening and such and, um, what you're saying about, you know, the what if it's wrong and the doctors, um, making their own decisions.  Um, I feel as though overall, um, having the AI there is a benefit even if, um, it - they have to change the way they work with it.  They have to learn how to use it and interpret its results. Um, and even if they - they do make mistakes from time to time, they already make mistakes.  And having, ah, the AI there to - as an additional thing to - to consult and to see what it says is helpful for these doctors to, um, what's the word - to make their own determination.*  ---  In Session 14 (areas for recommendations) jurors disagreed on what they meant when they discussed ‘access’.  *P4:                   So an easy way to, that I'm thinking of is I'm not sure if it's gonna help, but for person-centred care, it's kind of like a theory of approach.  You know, we want to make sure that the, you know, the person is considered and that can be the clinician's using this.  So when I'm looking after a patient, I might be making sure I'm taking into account the patient values.  So it's a bit of a theoretical aspect.  And the access part is more where are the barriers, where are the road blocks that are in place and the access to getting to these health services.  So we might have the same theoretical stance, but the access and equity sort of part is kind of the barriers and enablers of healthcare provision.==*  *P6:                   I think we've got different definitions of access.  What I think of access is different from yours*  *P3:                   Yes.  Yeah.  Yeah.  100%.  Absolutely.*  *P2:                   So, so what is your definition of access?  What was yours again?*  *P6:                   Well, availability.  It should be entitlement to everyone over to it.*  *P3:                   Basically, you might to have, um, the ability to.*  *P2:                   Okay, and that's where I say in the real world, no, it doesn't happen that way.*  *P6:                   You agree?  But it should.*  *P3:                   But we have to highlight it.  I think, yeah.  Okay.  Well, we'll work on the wording, but let's - I think ethical principles with person centred care is something we all agree on?* | **Yes, there is some evidence to suggest that this criterion is met**  No, this criterion is not met (either insufficient evidence or evidence does not show that criteria is met) | Also evidenced by recommendations that did not get 100% agreement. |
| **T** | 2.4.7. Transcripts show evidence of jurors exposing their assumptions | In session 13 (world café), a juror acknowledges how their personal experience of living in a rural area is affecting the things that they think are important  *P10:       ‑ ‑ ‑ and we can’t get, just if you’ve got a runny nose, it’s eight weeks pretty much until you can get an appointment … and then it’s a four hour drive just to get an MRI, or like there’s no facilities where I live to get that stuff, and if you’re crook, you will jump in the car for four hours, take the day off work and drive back, and they give you a referral to come back in three weeks.*  *P9:         Yeah.*  *P10:       Um, so that’s the rural remote side and that’s a bias that – that I’m coming from because that’s – that’s the reality*  ---  Also in Session 13, jurors discussed their tendency to focus on harms more than benefits.  *P1:         It’s hard not to think about a benefit and then like, you know ‑ ‑ ‑*  *P4:         The flip side.*  *P1:         ‑ ‑ ‑ then think about the other side of the coin, every benefit seems to have a harm.*  *P4:         And as humans we actually tend towards the negative than the positive, so it’s harder for us to focus on the positives as humans ‑ ‑ ‑*  *P1:         Cautious.  Yeah.*  *P4:         ‑ ‑ ‑ we focus easily on negatives, and we struggle to focus on positives, so* | **Yes, there is some evidence to suggest that this criterion is met**  No, this criterion is not met (either insufficient evidence or evidence does not show that criteria is met) | Second quote shows better evidence of this than first quote. |
| **T** | 2.4.8. Transcripts show evidence of jurors exploring uncertainties | There are many examples of jurors discussing uncertainties with one another. In session 13 (world café), jurors discuss how to ensure that AI systems work well for diverse populations in Autralia.  *P6:         I don't think they can introduce the AI until it's fully tested and trailed in - in society of a wider group of society.  They can't just test people in Sydney when you've got people in the outback that are going to be tested.*  *P12:       Well, yeah, I think inclusivity with all groups will impact.*  *P6:         Yeah.*  *P12:       So the testing has to be inclusive as well.  Yeah.*  *P6:         Well, how - you know, how - how does it work now like if they're trying to try the AI system.  Like are they using data for like testing that's already been done? Or are they starting the AI and then like test and then you're putting that data into a hardware? [[Jurors explored uncertainties]]*  *P12:       I imagine they would have to design something and then test it first and then improve and expand and improve and expand until they get to the point where they feel that this is ready to be released on a wider scale.*  ---  In session 7b, the jurors discuss uncertainties about existing regulatory structures in Australia and internationally  *“P7:         Ah, I - I just want to know, um, is there any regulation about AI in Australia and, ah, what - what we need, ah, to regulate, ah - ah, what - what kind regulation we need for AI in Australia and, ah, any, um, international organisation to do this job? Ah, I mean the international cooperation, ah, how to manage and share those data or manage, ah, how to use, ah, those patient data, ah, that that's, ah, maybe, ah, international organisation can do something so they can share more data and, ah, to make those kind of technology can be, um, benefited for everyone all around the world.*  *P6:         I was actually, yeah, that's a good point, {NAME REMOVED}.  I was - I was pleased to see, I think I wrote down the Australian Commission for Safety and Quality and Healthcare, and there's also the Australian Digital Health Agency.  Um, so it'd be interesting to know, does everyone have to report to them? Um, and how do they analyse what's good, what's bad, what - what can be used, what can't be used?”* | **Yes, there is some evidence to suggest that this criterion is met**  No, this criterion is not met (either insufficient evidence or evidence does not show that criteria is met) |  |
| **T** | 2.4.9. Transcripts show evidence of jurors weighing alternatives and trade-offs | In Session 14 (areas for recommendations), a group discusses whether their recommendation that AI should be accessible to all is practical and implementable. They note that there are practical challenges with infrastructure in rural areas  *“P6:                   I'm wondering whether, um, because, sorry, I'm thinking making some - making it able to be accessible is a hard thing to say in a recommendation.  So it must be accessible to all that, you know, have pragmatic complications everywhere.  But whether we had to change it to, you know, it needs to be considered, um, you know, is probably an easier way to recommend that because making sure it's gonna roll out to rural Australia is a hard task.*  *P3:                   Oh sure.*  *P2:                   And we have to really talk about the real world.  The real world is everyone is not going to be, we would - and you can't really, you can't really have it as a recommendation because realistically there's costs involved and you know, and there's no way.  I mean, if we're going to put a recommendation in, but we've gotta put something that is doable.  Something that's- - -*  *P3:                   Yeah enact-able*  ---  In session 13 (world café), the jurors became sceptical of whether governments would be overly focused on the economic benefits of AI at the expense of ensuring that the tool is used fairly. They discussed whether trading off fairness for economic benefit was ever worthwhile  *P6:         What - what matters more? The dollars or the people?*  *P15:       The dollars.  It's true, right?*  *P12:       It's a cynical response, but - - -*  *P15:       It is.  It's all about the money.*  *P12:       - - - that's society,*  *P15:       Isn't it? I hate that.  That's why I live out [rurally].  You can keep all your money.  It's down by the - - -*  *P12:       Same here.*  *…*  *P5:         It starts with a policy, but it ends up on a balance sheet.*  *P12:       Yes.  I like that saying.  I'll will try and remember that one.  Yeah.  But I mean, again, on the flip side, I seem to be playing the devil's advocate here, um, yeah, the dollars are important.  Um, because if we don't have those investors, we don't get their systems.  So if there's not money in it for someone, then they're not going to put the effort into designing and creating these systems for the people.  So, um, while, you know, I'm - I'm not for everyone just making a quick buck, unfortunately, we need them.  Otherwise there's not gonna be any money.  The government can't just keep sinking money into this.  The government does rely on those private, um, investors, entrepreneurs to put the money in to develop these programs.* | **Yes, there is some evidence to suggest that this criterion is met**  No, this criterion is not met (either insufficient evidence or evidence does not show that criteria is met) |  |
| **T** | 2.4.10. Transcripts show evidence of jurors providing justification for their viewpoints | In session 14 (areas for recommendations) the jurors discussed making a recommendation that clinicians are explicit about their use of AI. They justify this by highlighting the importance of clinicians taking ultimate responsibility for medical decisions  *P3: So mandatory reporting about how they use the AI and then, I suppose, ah, maybe what they found.  But yeah, how they used it really, and maybe why --*  *P1:                   I was just thinking it was just straightforward as like - - -*  *P3:                   The fact that they did use it.*  *P1:                   - - -this is like – … ‘this impacted my decision by X, like I thought it.  As in, I had to consider this all confirmed my findings.’*  *P3:                  ‘ Yes.  I agreed with the AI's findings.’*  *P4:                   Data on how these clinicians actually engage.*  *P3:                   Actually engage.*  *P1:                   And make sure that they're not being left to be autonomous.*  *P4:                   It's very important because it traces the responsibility, that's what you're doing*  ---  In a different group in session 14, the jurors discussed making a recommendation about an AI regulatory framework. They justified this by emphasising the importance of accountability, fairness, and equity.  *P1:         Yeah.  And the goal is, of course, having a regulatory framework is for account – to bring about accountability, quality.*  *P2:         Yep.*  *P1:         Um, fairness, what else?  What is the goal?*  *P3:         Uh, is to – you know, is to – I guess to reduce error – errors.*  *P1:         Yeah.*  *P3:         Uh, reduce - - -*  *P1:         [00:26:54] harm.*  *P3:         - - - [00:26:55] harms and, um, make sure the benefits are distributed.*  *P4:         To the people who – who – who need them.*  *…*  *P4:         I mean, the whole – the whole reason for the existence is to benefit the community and not – not people in positions of power.* | **Yes, there is some evidence to suggest that this criterion is met**  No, this criterion is not met (either insufficient evidence or evidence does not show that criteria is met) |  |
| **T** | 2.4.11. Transcripts show evidence of the jurors engaging with the evidence in their discussion | In session 14 (areas for recommendations), jurors discuss the conversation they had with Snr Prof Wendy Rogers about existing ethical guidelines for AI. They use this to inform their approach to setting ethical guidelines.  *P1:                   So a governing body sounds good.*  *P2:                   I forget what her name was, but the woman like expert, that was sitting here - - -*  *P3:                   Wendy Rogers?*  *P2:                   Yeah … with what she was saying about, ah, how there's just like a million different charters and stuff like that.  But the most effective thing at the end of the day and what they all sort of end up boiling down fitting into just being like, three, just like really instead of like specific points.  Literally just like guiding principles being like more theoretical ideas that have to fit within. Just like not harming patients.*  *P1:                   A high level ethical code rather than a specific - - -*  *P2:                   Like guidelines.*  *P1:                   Okay.*  *P3:                   Yeah, so those guiding principles would be high level rather than really nitty gritty.  You guys, you've got your own.*  --  Also in session 14, a separate group referred to a diagram about evidence-based practice that was shared by Prof Katy Bell in her evidence package.  *P3:                   Fair process, evidence-based practice, yeah.  Do you want me just to write these in - - -*  *P1:                   Yeah, yeah.  Just in one word for them.  Yeah.  Fair process.*  *P3:                   Fair process, um, evidence-based practice.*  *P5:                   Is that notion of, you know, evidence-based practice that triangle they showed in week one.  Being, it has to take into account the patient values, and if we're in ethics, we're already thinking about what's good for the patient.* | **Yes, there is some evidence to suggest that this criterion is met**  No, this criterion is not met (either insufficient evidence or evidence does not show that criteria is met) |  |

Final reflexive evaluation for **2.4 Quality of judgement.** Based on the evidence above, to what extent did the jury process satisfy this criterion?

| Strengths | Opportunities |
| --- | --- |
| - Even though not all jurors changed their mind, there was definite openness in the group to change - Jurors frequently referred to the evidence |  |

## 2.5: Perceived knowledge gains by members

|  | Review criteria | Evidence | Rating | Comment |
| --- | --- | --- | --- | --- |
| **Q** | 2.5.1. Jurors agreed that “the jury has changed my awareness of different points of view about [POLICY AREA]” | 93% of jurors agreed that participating in the jury changed their awareness of different points of view about AI in diagnosis and screening | **Yes, there is some evidence to suggest that this criterion is met**  No, this criterion is not met (either insufficient evidence or evidence does not show that criteria is met) |  |
| **Q** | 2.5.2. Were there any changes between timepoints in item “how knowledgeable are you about [POLICY ISSUE]” | At T1, 11% of jurors (3/28) were knowledgeable/very knowledgeable about AI. This increased to 86% (24/28) by T3. | **Yes, there is some evidence to suggest that this criterion is met**  No, this criterion is not met (either insufficient evidence or evidence does not show that criteria is met) |  |
| **Q** | 2.5.3. 2.5.2. Were there any changes between timepoints in the item(s) about jurors’ attitudes toward the policy issue? | At T1, 75% of jurors supported/strongly supported development of AI and 69% supported/strongly supported its use in healthcare. Both increased to 93% by T3. | **Yes, there is some evidence to suggest that this criterion is met**  No, this criterion is not met (either insufficient evidence or evidence does not show that criteria is met) |  |

Final reflexive evaluation for **2.5 Perceived knowledge gains by members.** Based on the evidence above, to what extent did the jury process satisfy this criterion?

| Strengths | Opportunities |
| --- | --- |
| People’s understanding of different points of view changed, and people’s perceived knowledge increased. | Changes in self-perceived knowledge and level of support may not reflect actual increases in knowledge or changes in views. Potential opportunity to test knowledge in other ways? |

## 2.6: accessibility and equality of opportunity to speak

|  | Review criteria | Evidence | Rating | Comment |
| --- | --- | --- | --- | --- |
| **Q** | 2.6.1. Jurors agreed that “there were equal opportunities for all jurors to express their views”  CHANGE THIS “there were opportunities for all jurors to express their views” | At T2, 93% (26/28) jurors agreed that there were equal opportunities for all jurors to express their views. This decreased slightly to 89% (24/27) at T3 (one juror did not respond). | **Yes, there is some evidence to suggest that this criterion is met**  No, this criterion is not met (either insufficient evidence or evidence does not show that criteria is met) | Interesting that this decreased slightly between timepoints. Some juror comments in the eval questionnaire said that some people got to talk more than others. Potential opportunity to improve access to opportunities to speak in the face-to-face components. |
| **T** | 2.6.3. The transcripts show evidence that modifications were made to the process, where (and if) jurors requested them | During the ‘areas for recommendation’ session, Stacy noticed that jurors were having a hard time coming up with recommendations within the allotted time.  Q2:        I’m getting a sense that there’s too much time pressure on this task.**  P2:         Yeah.  **Q2:        Other people are feeling frustrated - - -**  P1:         Yes.  **Q2:        - - - about the time pressure, am I right?**  P2:         Yeah.  P3:         Yep.  **Q2:        So you know what?  Do you guys agree?  Too much time pressure?  Yeah.  So I really don’t want you to feel that way.  We were – so we were going to try and swap groups around so that you had more cross-fertilisation, but I think it’s actually more important to give you depth.**  P2:         Yep.  **Q2:        And the support of working with the same group.  So with your permission, what we might do is change this – change the way we’re doing the task, so that you work on this until lunch and then the research team will work together over lunch to organise your areas of recommendation into clusters for you, so that you don’t have to do that and that will get us a bit of time and then you can tell us after lunch, whether we’ve done it right.  Um, but it will give you more time to work on what you want to include in your recommendations.  Does everyone agree that that’s a good change to the process?**  ---  Later, during the pitch, some jurors felt that the process of giving feedback on the recommendations was not working. They suggested putting the feedback on the wall for jurors to make written comments.  ***Q: So, I'm trying to think about how to take all this on board and potentially abandon this. ‘Cause if it's not working for the group, then we shouldn't keep going. Right? So, we've got a couple of options. P2?***  *P2: I still would like to hear the recommendations because I think everybody's really done a great job and I think it deserves the opportunity for us to be able to say, this is what we've done. And then maybe from there you can put 'em on the wall, give us a few minutes to read it and then maybe circle back and go, do you have any comments on them? Ah, as such you've gone and walked them through.*  ***Q: Yeah, I was thinking of something like that too. What do other people think? Yeah? Okay, so here's how we might proceed, and you tell me if you like this. We'll keep going with the reading out the recommendations; once every group has had two, we'll get you to put them as they stand on the wall with a sticky dot.*** | **Yes, there is some evidence to suggest that this criterion is met**  No, this criterion is not met (either insufficient evidence or evidence does not show that criteria is met) |  |

Final reflexive evaluation for **2.6 accessibility and equality of opportunity to speak.** Based on the evidence above, to what extent did the jury process satisfy this criterion?

| Strengths | Opportunities |
| --- | --- |
| Jurors had the opportunity to comment on the process and make modifications where required. | In a perfect world, it would be good to log the distribution of speaking time to understand whether some jurors dominated discussions. |

## 2.7: respect and mutual comprehension

|  | Review criteria | Evidence | Rating | Comment |
| --- | --- | --- | --- | --- |
| **Q** | 2.7.1. Jurors agreed that “jurors were listening to each other and allowing each other to speak” | At T2, 96% of jurors agreed. At T3, 100% of jurors agreed. | **Yes, there is some evidence to suggest that this criterion is met**  No, this criterion is not met (either insufficient evidence or evidence does not show that criteria is met) |  |
| **Q** | 2.7.2. Jurors agreed that “I feel that I was listened to by the facilitator” | At T3, 96% (27/28) jurors agreed. | **Yes, there is some evidence to suggest that this criterion is met**  No, this criterion is not met (either insufficient evidence or evidence does not show that criteria is met) | Facilitator occasionally had to intervene to remind jurors to give their peers opportunities to share their views. |
| **Q** | 2.7.3. Jurors agreed that “I felt that my opinions were respected by the group” | At T3, 100% of jurors agreed. | **Yes, there is some evidence to suggest that this criterion is met**  No, this criterion is not met (either insufficient evidence or evidence does not show that criteria is met) |  |
| **T** | 2.7.4. Transcript shows that jurors encouraged one another to share their views | In the speed dialogue with Prof Rogers, one juror acknowledges that they have been speaking too much and lets other jurors ask questions:  *Prof Wendy Rogers: … But I'm sure you've got questions, so happy to answer.*  *P1:            I've got lots.  [laughter]*  *P2:            Anything you can think of {P1}? Which obviously - - -*  *P1:            I can, but you know what.  I've been talking a lot and a lot of other people haven't been, so I'm actually gonna be quiet.  Sorry.*  *P2:            Who feels they haven't had a chance yet?*  ------  In Session 14 (areas for recommendations), jurors decide on a process for how each of them will share their individual recommendations, and how they will decide fairly as a group to prioritise them:  *P3:                   But do we wanna have like a democratic process where like, everyone shares, like how we gonna - what's the process we're gonna share?*  *P4:                   If we've got some that, you know, resonate from us, then maybe be focus on and develop maybe a hierarchy of our importance.  That's one that we've all got something to say about. Yeah.*  *P6:                    I suggest that we just go through each of our - - -*  *P1:                   Yeah, perfect.*  *P6:                   - - -yeah, recommendations go through.*  *P1:                   Yep* | **Yes, there is some evidence to suggest that this criterion is met**  No, this criterion is not met (either insufficient evidence or evidence does not show that criteria is met) |  |
| **T** | 2.7.5. Transcript shows that jurors considered one another’s views | In session 13 (harms) one juror (P3) helps another juror (P5) clarify a point that they have having trouble articulating.  *P5:         Because I think they — see under normal circumstances and it's not technology getting involved, human interest, systems and processes that the society establishes to provide checks and balances.  But then when there is something introduced at a massive scale, then we have to start thinking about saying it's not just a question of the best technology, it is also about convincing others.  And there are multiple players as we just established in the stakeholders that we are all concerned about doing a better patient care.  But the problem of course is that each one has an interpretation of it and therefore, you know, doctor or nurse or a clinician or radiologist or somebody else …  And that's where my  biggest concern is. … The technological aspects of the scientific paradigm for technological aspects is quite different to the human paradigm and the human interaction.  And the human engagement is so different that you can have a very sophisticated system.*  *P2:         Almost puts you in a box.*  *P5:         Put you in a box.*  *P2:         Yeah.*  *P5:         Exactly.  It's a box.*  ***Q1:        Could you think of a way to put that down for the next group? …*  *P3:         Maybe something like a — a due diligence of that type of thing where someone overlooks [oversees] what you're talking about and the whole system before it actually is introduced.  I think that's what you're trying to say with everything.  The data, the program, the whole thing.  Everyone there is a body that overlooks [oversees] it.  Like maybe a policy, putting a policy together.*  *P5:         Social impact — social impact and social — and, you know, economic, social, political administrative management plan.*  *P3:         Yeah.*  *----------*  In another group in session 13 (bias and fairness), another group of jurors help one another clarify a point  *P15:       …  This notion of transparency being important and embedded everywhere, um, because how that's going to prevent bias from their outcomes?  But it's also more than just transparency.  It's having the accountability with that transparency.  So having some point of the - in the system being accountable for their actions or inactions is probably going to help this as well.  And I'm just trying to figure out how to word that…*  *P13:       Yeah.*  *P12:       Yeah.  Yeah.*  *P16:       Accountability throughout the system, right?  So it's not just internal regulation.*  *P15:       Exactly.  Yeah.  Yeah.* | **Yes, there is some evidence to suggest that this criterion is met**  No, this criterion is not met (either insufficient evidence or evidence does not show that criteria is met) |  |

Final reflexive evaluation for **2.7 Respect and mutual comprehension.** Based on the evidence above, to what extent did the jury process satisfy this criterion?

| Strengths | Opportunities |
| --- | --- |
| - Jurors generally felt heard and respected by their fellow jurors and by the facilitator |  |

## 2.8: Free decision-making and response

|  | Review criteria | Evidence | Rating | Comment |
| --- | --- | --- | --- | --- |
| **D** | 2.8.1. The final report explains how democratic decision-making rules (e.g. consensus, majority rule, ranking) were used to generate the final recommendations | Page 411 of the report says:  *“The jury then drafted recommendations in their own words in*  *each of the revised categories, working in self-selected working*  *groups (four to seven people) and drawing on written records*  *of their earlier discussions. All jurors provided input through*  *iterative cycles of plenary feedback, re-drafting, and voting.*  *A recommendation was included in the report if at least 24*  *jurors supported it”* | **Yes, there is some evidence to suggest that this criterion is met**  No, this criterion is not met (either insufficient evidence or evidence does not show that criteria is met) |  |
| **D** | 2.8.2. Documents show that jurors had the opportunity to express alternative views in a minority report. | From moderators guide:  “We are looking for a supermajority of people who can at least live with recommendations – but there can be a minority report if really needed”  …  “If there are any [recommendations] for which disagreement can’t be resolved – do we need a minority report?  If there is a minority report, those people might need to go off to write the minority report”  …  “If minority report, that needs to be presented to the whole jury – needs to be in the same format, recommendations and reasons “ | **Yes, there is some evidence to suggest that this criterion is met**  No, this criterion is not met (either insufficient evidence or evidence does not show that criteria is met) |  |
| **Q** | 2.8.3. Jurors agreed that “it was clear how we were meant to arrive at a decision” | 93% (26/28) of jurors agreed that ‘it was clear how we were meant to arrive at a decision’ | **Yes, there is some evidence to suggest that this criterion is met**  No, this criterion is not met (either insufficient evidence or evidence does not show that criteria is met) |  |
| **T** | 2.8.5. Transcripts show that jurors had the final say over the wording of the recommendations | In the final wordsmithing and voting on the recommendations, the facilitator copied wording from the ‘recommendations’ forms that jurors submitted. The facilitator then went through a wordsmithing process with the jurors and checked wording with the jurors:  ***Q: There is. Yeah. So what does it say on the form? So the - so the recommendation is that the chair - the chair is independent from health and investors to avoid bias. So the - I think that means the health system and investors to avoid - avoid bias.***  *P2: Can you say that again please?*  ***Q: So we recommend the board is chaired independent of the health system and investors to avoid bias. Um, the working group is that - was that the intention?***  *P3: Yes.*  ***Q: Yes?***  *P3: Yeah. Independent of.*  ***Q: Independent of, yeah?***  *P2: Yeah.*  ***Q: Yeah. Is that - the working group - is that actually what was intended?***  *P4: Yes.*  ----  In this same session, when jurors made suggestions or clarifiications for the recommendations, edits were made in the recommendations document live.  ***Q: Okay. The third one about clinical training. We recommend that there should be a reporting process. So maybe that there should be a mandatory reporting process to the appropriate governing body or bodies. Is that okay? Is that - is that consistent with the intention for the drafters?***  *P12: Yeah.*  ***Q: Yeah. Um, such a reporting process should include unfavourable outcomes, performance misuse, and any benefits to the patients, clinicians and healthcare systems. P14?***  *P14: Sorry. Um, the only thing – I was drafting, I was considering mandatory reporting being like a verifiable conduct like for healthcare professions. So I wondered why we purposely chose to move that verdict behind? So we want to make it explicit that we, um, there needs to be a process of reporting, not a mandatory reporting process.*  ***Q: Oh, okay. Oh, I'm sorry. My - my apologies. Yeah. Okay. So - so can we say we recommend that? Can we take out there should be and just say we recommend that a reporting process be made mandatory. Is that okay? Yeah. Is that more consistent?***  *P2: Yeah.*  *P11: Yeah. Like a usage reporting, whatever, just to make it clear that it's like not just in incidents.* | **Yes, there is some evidence to suggest that this criterion is met**  No, this criterion is not met (either insufficient evidence or evidence does not show that criteria is met) |  |

Final reflexive evaluation for **2.8 Free decision-making and response.** Based on the evidence above, to what extent did the jury process satisfy this criterion?

| Strengths | Opportunities |
| --- | --- |
| - Democratic decision rules were used to formulate the recommendations - Jurors had the opportunity to draft a minority report, although this was not required. - Jurors had the final say over the wording of the recommendations. |  |

## 2.9: respect for members’ privacy

|  | Review criteria | Evidence | Rating | Comment |
| --- | --- | --- | --- | --- |
| **D** | 2.9.1. The jury had processes in place to prevent jurors' identity from being revealed where the juror did not want it to be (e.g. processes preventing photos being taken unless juror has given informed consent, anonymisation processes on evaluative surveys) | We had several processes in place to ensure that jurors did not have to share their identity if they did not want to:   - Only first names were shared on name tags and in Zoom names. Surnames were removed. - Jurors all had the option to sign a form allowing us to photograph them. If they did not sign the form, they were not photographed. - Unique numbers were used to identify jurors’ evaluation forms so that the forms did not identify them by name - Observers signed a document saying that they must maintain the confidentiality of jurors, including not taking photos or videos of the process, or taking notes that identify jurors. | **Yes, there is some evidence to suggest that this criterion is met**  No, this criterion is not met (either insufficient evidence or evidence does not show that criteria is met) |  |
| **D** | 2.9.2. Any observers of the jury were instructed to refrain from interfering in the jury processes | Observers all signed an agreement with 10 obligations, including:  “5) Observers should not approach the jury or interrupt the jury process.”  “8) If, during the jury process, jurors have questions for particular observers, the facilitators may invite an exchange between the jurors and those observers.”  “9) There may be some sessions where facilitators ask observers to leave the room. If this occurs we will make the request discretely and explain why, and will let observers know when they can return.” | **Yes, there is some evidence to suggest that this criterion is met**  No, this criterion is not met (either insufficient evidence or evidence does not show that criteria is met) |  |

Final reflexive evaluation for **2.9 Respect for members’ privacy.** Based on the evidence above, to what extent did the jury process satisfy this criterion?

| Strengths | Opportunities |
| --- | --- |
| - There were processes in place to protect the privacy of jurors - Observers were instructed not to interfere |  |
